# Acetone utilization by sulfate-reducing bacteria: draft genome sequence of *Desulfococcus biacutus* and a proteomic survey of acetone-inducible proteins

**DOI:** 10.1186/1471-2164-15-584

**Published:** 2014-07-11

**Authors:** Olga B Gutiérrez Acosta, David Schleheck, Bernhard Schink

**Affiliations:** Department of Biology and Konstanz Research School Chemical Biology, University of Konstanz, D-78457 Konstanz, Germany

**Keywords:** *Desulfococcus biacutus*, Acetone activation, Genome sequencing, Carbonylation, Thiamine diphosphate

## Abstract

**Background:**

The sulfate-reducing bacterium *Desulfococcus biacutus* is able to utilize acetone for growth by an inducible degradation pathway that involves a novel activation reaction for acetone with CO as a co-substrate. The mechanism, enzyme(s) and gene(s) involved in this acetone activation reaction are of great interest because they represent a novel and yet undefined type of activation reaction under strictly anoxic conditions.

**Results:**

In this study, a draft genome sequence of *D. biacutus* was established. Sequencing, assembly and annotation resulted in 159 contigs with 5,242,029 base pairs and 4773 predicted genes; 4708 were predicted protein-encoding genes, and 3520 of these had a functional prediction. Proteins and genes were identified that are specifically induced during growth with acetone. A thiamine diphosphate-requiring enzyme appeared to be highly induced during growth with acetone and is probably involved in the activation reaction. Moreover, a coenzyme B_12_- dependent enzyme and proteins that are involved in redox reactions were also induced during growth with acetone.

**Conclusions:**

We present for the first time the genome of a sulfate reducer that is able to grow with acetone. The genome information of this organism represents an important tool for the elucidation of a novel reaction mechanism that is employed by a sulfate reducer in acetone activation.

**Electronic supplementary material:**

The online version of this article (doi:10.1186/1471-2164-15-584) contains supplementary material, which is available to authorized users.

## Background

Aerobic and nitrate-reducing bacteria can utilize acetone for growth through activation of the acetone molecule by an endergonic carboxylation reaction to form acetoacetate as a first reaction product [1–3]. For example, the respective enzyme in *Xanthobacter* strain Py2, acetone carboxylase (EC 6.4.1.6) [1, 4], activates acetone with CO_2_ to form acetoacetate at the expense of ATP. ATP is hydrolyzed to AMP plus pyrophosphate which is later hydrolyzed to two phosphates, hence, the reaction consumes two ATP equivalents if the regeneration of ATP from AMP is taken into account. The acetone carboxylase of the nitrate reducer *Aromatoleum aromaticum* [2] activates acetone with bicarbonate at the expense of two ATP that are hydrolyzed to two AMP plus four phosphate, hence, effectively at the expense of even four ATP equivalents. Aerobic and nitrate-reducing bacteria can gain sufficient energy from the oxidation of acetoacetate to sustain this energy input required for the initial carboxylation of acetone, in addition to the energy input required to sustain their growth.

Acetone activation by the strictly anaerobic, dissimilatory sulfate-reducing bacteria (SRB) has not been studied in detail yet, and it is unknown how these bacteria perform the endergonic activation reaction. The overall energy derived from acetoacetate oxidation with sulfate as electron acceptor (that is, about two to three mol ATP per mol acetoacetate), appears to be hardly sufficient to support growth and activation of acetone *via* any of the carboxylase reactions described above. Up to date, two acetone-utilizing SRB are known, the deltaproteobacteria *Desulfobacterium cetonicum* [5] and *Desulfococcus biacutus* strain KMRActS [6]; the latter bacterium has been isolated from an anaerobic sludge digestor of a wastewater treatment plant.

Recently, we proposed that *D. biacutus* activates acetone in a carbonylation reaction to form acetoacetaldehyde [7]. The reaction was followed in cell-free extracts of acetone-grown *D. biacutus* and requires ATP as a cofactor, and CO rather than CO_2_ as a co-substrate [7]. The reaction product acetoacetaldehyde was trapped as the dinitrophenyl-hydrazone derivative and identified by mass spectrometry after a reaction of acetone, CO, ATP, and dinitrophenylhydrazine with cell-free extracts [7]. The reaction could not be observed with cell-free extract of butyrate-grown *D. biacutus*, hence, the proposed acetone-activating enzyme appeared to be inducible*.* However, this novel reaction, and particularly the postulated enzyme(s) and gene(s) involved, remained undefined.

Complete genome sequences of several SRB have become available in the recent years, for example, of the unclassified deltaproteobacterium strain NaphS2 that is able to utilize naphthalene [8], of *Desulfobacula toluolica* strain Tol2 that grows with aromatic compounds [9], of *Desulfobacterium autotrophicum* strain HRM2 that is able to utilize fatty acids [10], of *Desulfatibacillum alkenivorans* AK-01 that utilizes hexadecane [11], and of *Desulfotalea psychrophila* that uses lactate and alcohols [12], as well as of *Desulfotomaculum acetoxidans* [13] and of a *Desulfarculus baarsii* strain [14]. Only one genome of a member of the genus *Desulfococcus* has been made available thus far, i.e. *Desulfococcus oleovorans* strain Hxd3 (JGI project id: 4002948) which utilizes C_12_-C_20_ alkanes for growth [15].

In the present paper, we report the draft-genome sequencing and annotation of the first genome of an acetone-degrading, sulfate-reducing deltaproteobacterium, i.e., of *Desulfocccus biacutus* strain KMRActS, and hence, of a second member of the genus *Desulfococcus*. The genome information was used in a differential-proteomics approach to identify genes that are specifically expressed during growth with acetone, in comparison to growth with butyrate. Candidate genes were identified that could be involved in the activation and degradation of acetone in *D. biacutus*.

## Results and discussion

### General features of the *Desulfocccus biacutus*genome sequence

Genomic DNA of *D. biacutus* KMRActS was extracted and submitted to whole-genome shotgun sequencing using a Roche GS FLX + system (see Methods). Sequencing and assembly resulted in a genome of a total size of 5,242,029 bp distributed over 159 contigs. The contigs were annotated *via* the Integrated Microbial Genomes (IMG) pipeline (Table 1). In total, 4773 open reading frames (ORFs) were predicted, of which 4708 (98.6%) were protein-encoding genes, 3520 (73.8%) genes coding for proteins with a predicted function, and 1128 (23.6%) were attributed to encode transmembrane proteins; only one set of rRNA genes was found.

**Table 1:** **Statistics of the IMG genome annotation of** ***Desulfococcus biacutus***

	Number	% of Total
**DNA, total number of bases**	5242029	100.00%
DNA coding number of bases	4646037	88.63%
DNA G + C number of bases	3055509	58.29%
**DNA scaffolds**	159	100.00%
CRISPR Count	8	
**Genes total number**	4773	100.00%
Protein-encoding genes	4708	98.64%
RNA genes	65	1.36%
rRNA genes	3	0.06%
5S rRNA	1	0.02%
16S rRNA	1	0.02%
23S rRNA	1	0.02%
tRNA genes	51	1.07%
Other RNA genes	11	0.23%
Protein-encoding genes with function prediction	3520	73.75%
Protein coding genes coding transmembrane proteins	1128	23.63%
Protein coding genes connected to transporter classification	606	12.70%

In direct comparison to its closest genome-sequenced relative, *Desulfococcus oleovorans* strain Hxd3, the *D. biactus* genome appeared to be larger (5.24 Mb vs. 3.94 Mb, respectively; 4773 vs. 3320 ORFs) and, most strikingly, contained a much higher number of assigned transporter genes (606 candidate genes) compared to *D. oleovorans* (299 candidates); for example, more candidates of the ABC-superfamily (TC:3.A.1) (218 in *D. biacutus* vs. 77 in *D. oleovorans*), MFS-superfamily (TC:2.A.1) (17 vs. 8), and TRAP-family (TC:2.A.56) (21 vs. 12). Furthermore, a higher abundance of genes for signal transduction and regulation was found, e.g., for signal transduction histidine kinases (COG4585 and 0642) (43 candidates in *D. biacutus* vs. 18 in *D. oleovorans*), CheY-like response regulators (COG2197 and 2204) (73 vs. 43), and PAS-containing transcriptional regulators (COG3829) (11 vs. 5). In total, eight CRISPR elements were predicted for *D. biacutus*, and two for *D. oleovorans*. In contrast, the genome of *D. biacutus* harbors less candidate genes for fatty-acid metabolism, e.g., for acyl-CoA dehydrogenases (COG1960) (12 candidates in *D. biacutus* vs. 25 in *D. oleovorans*), enoyl-CoA hydratases (COG1024) (8 vs. 16), and acyl-CoA acetyl-transferase (COG0183) (8 vs. 13), with the exception of acyl-CoA synthetase candidates (COG0318 and 1042) (27 vs.18). The *D. biacutus* genome annotation also indicated a lower abundance of benzoyl-CoA reductase candidates (COG1775) (4 vs. 10 candidates in *D. oleovorans*). No valid candidate genes for *alpha* subunits of benzyl-, alkyl-, or naphthylsuccinate synthase (searching with BssA [O87943], AssA1 [B8FEM4] and NsmA [D2XBH8]) were found in either the *D. biacutus* or *D. oleovorans* genome, but glycyl-radical enzyme candidate genes were found that most likely represent *alpha*-subunit genes for pyruvate formate lyases (e.g., DebiaDRAFT_01145). Finally, we found no valid gene candidates for acetone carboxylase in the *D. biacutus* genome (see below).

### Differential proteomics approach

We aimed at an identification of all proteins that are specifically synthesized in *D. biacutus* during growth with acetone, in comparison to cells grown with butyrate. Therefore, the annotated draft-genome sequence of *D. biacutus* was used to generate a reference database for peptide mass fingerprinting (PMF). Soluble proteins and membrane proteins were analyzed separately by two-dimensional (2D) and 1D-PAGE, respectively (see Methods), and protein bands or spots of interest were excised for PMF identification. A differential total proteome analysis (Orbitrap-MS analysis) of all proteins in crude extracts (soluble and membrane proteins) was also performed in order to confirm and expand on the identifications made by the gel-based proteomics approach; the results can be found in the Additional files 1: Table S1 and Additional file 2: Table S2 published with this article.

### Proteins that were specifically formed during growth with acetone

**Figure 1:**
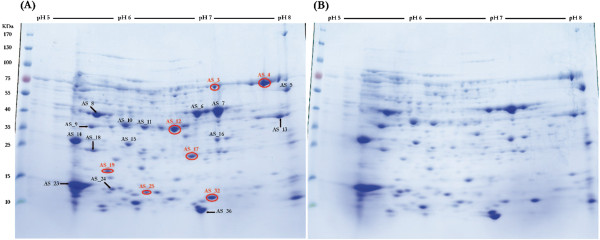
**2D-PAGE analysis of soluble proteins produced during growth with (A) acetone and (B) butyrate.** The protein spots that were identified by peptide mass fingerprinting (see Tables 2 and 3) are labeled in the acetone gel. Acetone-induced proteins are marked in red. M: molecular mass marker.

**Figure 2:**
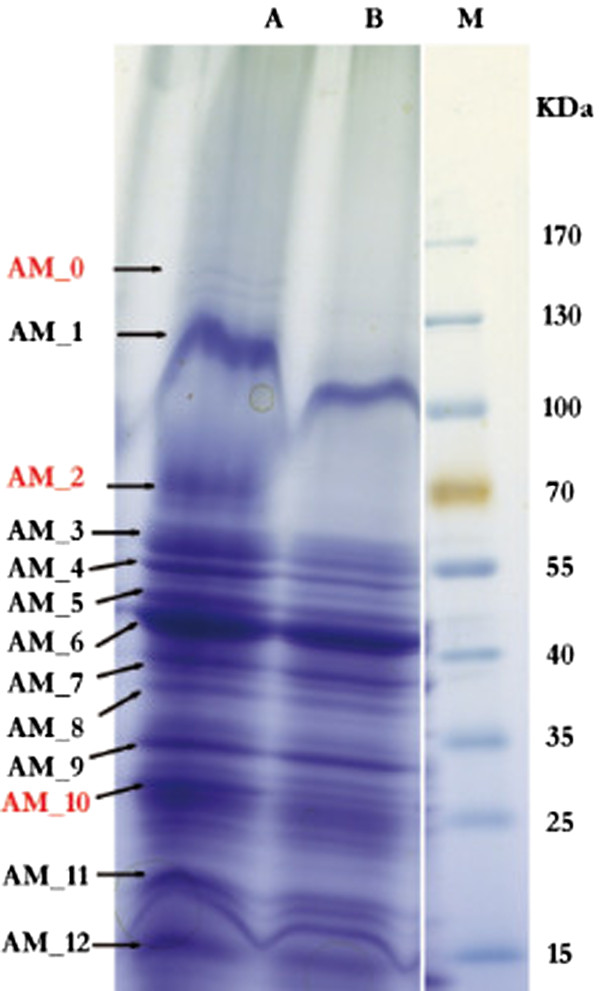
**1D-PAGE analysis of solubilized membrane proteins of acetone- (A) and butyrate-grown (B)** ***D. biacutus*** **cells (75 μg total protein each).** The protein bands identified by peptide mass fingerprinting (see Tables 2 and 3) are indicated. M, molecular mass marker.

**Figure 3:**
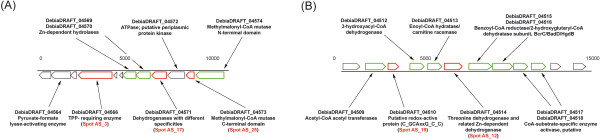
**Schematic representation of the two gene clusters (A and B) in** ***D. biacutus*** **harboring genes that appeared to be specifically induced during growth with acetone.** Their locus tags in the IMG genome annotation, the corresponding spot numbers on the 2D-PAGE gels, and a description of their annotation are indicated. The acetone-inducible genes identified *via* the differential 2D-gel-based analysis are labeled in red, and further genes that were identified in a differential total proteome analysis are labeled in green.

Proteins formed specifically during growth with acetone were separated by 2D-PAGE for the soluble protein fraction (Figure 1A,B) and by 1D-PAGE for the membrane fraction (Figure 2). The gels showed seven prominent spots in the cytoplasmic and three in the membrane fraction that were visible only on gels of acetone-grown cells but not on the gels of butyrate-grown cells (as indicated in Figures 1A,B and 2), and were excised and identified (Table 2). Furthermore, fifteen prominent cytoplasmic and five membrane-derived proteins observed on both gels, hence constitutively expressed proteins, were also excised and identified by PMF (Table 3). Five of the genes encoding the seven apparently acetone-inducible, soluble proteins (2D-PAGE spots) were found to be located on short contigs (app. 15 kb each) termed gene cluster A and B (see Figure 3A,B).

**Table 2:** **Identification of proteins observed specifically in extracts of acetone-grown cells of** ***D. biacutus*** **(see Figures** 1 **and** 2 **)**

Spot Id.	IMG locus tag (DebiaDRAFT_)	Annotation	Score
AS_3	04566	^§^Thiamine diphosphate-requiring enzyme	652
AM_2			282
AS_4	03619	^§^Adenosine phosphosulfate reductase *alpha* subunit	1595
AS_12	04514	^§^Zn-dependent dehydrogenases (threonine dehydrogenase)	970
	04509	^§^Acetyl-CoA acetyltransferase	200
AS_17	04571	^§^Dehydrogenases with different specificities (related to short-chain alcohol dehydrogenases)	1944
AS_19	04510	^§^Putative redox-active protein (C_GCAxxG_C_C)	1035
AS_25	04573	^§^Methylmalonyl-CoA mutase C-terminal domain	1315
AS_32	01796	Desulfoferredoxin	449
AM_0	04339	^§^Pyruvate:ferredoxin (flavodoxin) oxidoreductase	335
AM_10	03042	^§^Predicted NADH:ubiquinone oxidoreductase, subunit RnfG	159

^§^Proteins that were also found by Orbitrap analysis.

**Table 3:** **Identification of proteins common in extracts of acetone- and butyrate-grown cells of** ***D. biacutus*** **(see Figures** 1 **and** 2 **)**

Band Id.	IMG locus tag (DebiaDRAFT_)	Annotation	Score
AS_5	02387	^§^Formyltetrahydrofolate synthetase	1027
	03619	^§^Adenosine phosphosulfate reductase *alpha* subunit	343
	03447	^§^NAD(P)H-nitrite reductase	314
AS_6	03586	^§^ATP sulphurylase	1338
AS_7			1454
AS_8	04385	^§^Sulfite reductase *alpha* subunit (dissimilatory type)	814
AS_9	00156	^§^ABC-type amino acid transport/signal transduction systems periplasmic component/domain	1367
AS_10	02798	ABC-type amino acid transport/signal transduction systems periplasmic component/domain	1844
AS_11	01292	ABC-type amino acid transport/signal transduction systems periplasmic component/domain	1514
	03292	^§^Acyl-CoA dehydrogenase	326
AS_13	04384	^§^Sulfite reductase *beta* subunit (dissimilatory type)	682
AS_14	01640	^§^Pterin binding enzyme	771
AS_15	02722	^§^ABC-type amino acid transport periplasmic component	1168
AS_16	00168	^§^NAD-dependent malate dehydrogenase	1411
	02722	^§^ABC-type amino acid transport/signal transduction systems periplasmic component/domain	245
AS_18	04513	^§^Enoyl-CoA hydratase/carnithine racemase	847
	04490	ABC-type amino acid transport/signal transduction systems periplasmic component/domain	730
	01784	^§^Enoyl-CoA hydratase/carnitine racemase	467
	03805	^§^Short-chain dehydrogenases of various substrate specificities	417
AS_23	03620	^§^Adenosine phosphosulfate reductase *beta* subunit	535
AS_24			340
AS_36	03129	Uncharacterized conserved protein	635
AM_1	01638	^§^CO dehydrogenase/CO-methylating acetyl-CoA synthase complex *beta* subunit	588
AM_3	00010	^§^Acetyl-CoA carboxylase, carboxyltransferase component (subunits alpha and beta)	397
AM_4	03347	^§^Proton translocating ATP synthase, F1 *alpha* subunit	811
AM_5	03345	^§^ATP synthase, F1 *beta* subunit	1146
AM_6	00583	Hypothetical protein	700
AM_7	03345	^§^ATP synthase, F1 *beta* subunit	204
AM_8	03346	^§^ATPsynthase, F1 gamma subunit	651
AM_9	01843	^§^5,10-methylenetetrahydrofolate reductase	155
AM_11	03348	^§^ATP synthase, F1 *delta* subunit	607
AM_12	03344	^§^ATP synthase, F1 *epsilon* subunit	447

^§^Proteins that were also found by Orbitrap analysis.

The PMF of prominent spot AS_3 (Figure 1A) corresponded to locus tag (ORF) DebiaDRAFT_04566 (Table 2) in gene cluster A (Figure 3A) (in the following, the locus tag prefixes, DebiaDRAFT_, are omitted). ORF 04566 was also identified by the PMF of an acetone-inducible protein that was observed by 1D-PAGE in the membrane fraction as band AM_2. Notably, the preparation of the membrane fraction involved four washing steps, hence, it is unlikely that band AM_2 resulted from a soluble-protein contamination of the membrane preparation. Moreover, when comparing the intensity of the 2D-PAGE spot (AS_3) and 1D-PAGE band (AM_2) it appears that this enzyme is represented more prominently in the membrane than in the soluble protein fraction. However, for this protein no transmembrane helices or signal peptide sequences were predicted by IMG. Gene 04566 is annotated to encode a thiamine diphosphate (TDP)-requiring enzyme (COG0028; acetolactate synthase/pyruvate dehydrogenase [cytochrome]/glyoxylate carboligase/phosphonopyruvate decarboxylase). The identification of this TDP-enzyme and its induction during growth with acetone was confirmed in a replicate 2D-PAGE, when starting from an independent growth experiment (not shown), and by the total proteome analysis (Orbitrap-MS; see Additional file 1: Table S1).

The detection of this TDP enzyme suggests that TDP might play a key role as a cofactor in the initial acetone activation. TDP is known to participate in carbon-carbon bond-forming or bond-breaking reactions, especially with those substrates that contain a carbonyl group [16–21]. In a separate study employing a fluorescent ATP analogue as probe [22], we could show that ATP is converted to AMP plus pyrophosphate, and that this reaction is stimulated by the presence of TDP. One could speculate that either the central carbon of (activated?) acetone is bound to the ilyl carbon of TDP for the subsequent carbonylation, or that TDP first binds the CO before the carbonylation of activated acetone.

Two genes that are located on the same contig, that is, in gene cluster A together with the TDP-enzyme gene 04566 (Figure 3A), were found to encode other proteins that were specifically induced during growth with acetone. One is ORF 04571, identified by the prominent spot AS_17 (Figure 1A) which is annotated to encode a short-chain dehydrogenase/reductase protein (Table 2); these types of dehydrogenases/reductases (COG1028) possess at least two domains, one that binds NAD^+^/NADH or NADP^+^/NADPH, and another one that determines the substrate specificity and catalysis. This enzyme might represent an NAD^+^-dependent dehydrogenase that oxidizes acetoacetaldehyde to acetoacetyl-CoA [7]. The identification and induction of this gene during growth with acetone was also confirmed by the total proteome analysis (see Additional file 1: Table S1). The other acetone-induced gene in cluster A is ORF 04573, identified by the prominent protein spot AS_25 (Figure 1A), which is annotated as the cobalamin (B_12_)-binding subunit (COG2185) of a methylmalonyl-CoA mutase enzyme complex (Table 2). This gene identification and the inducible expression was also confirmed by the total proteome analysis, in which also the N-terminal methylmalonyl-CoA mutase subunit (gene 04574) was identified (see Additional file 1: Table S1). This enzyme complex performs various types of reactions at the cobalt-carbon bond of its B_12_-cofactor, e.g., the relocation of the carboxyl-CoA residue in the interconversion of methylmalonyl-CoA and succinyl-CoA. Such a cobalamin-dependent step might play a role in acetone activation by *D. biacutus* if the (activated?) acetone is carbonylated at the central carbon atom to form first a branched-chain intermediate which subsequently has to be isomerized (linearized).

A very prominent, apparently acetone-inducible protein, spot AS_12 (Figure 1A), identified ORF 04514 in gene cluster B (Figure 3B); the identification and induction of this gene during growth with acetone was also confirmed by the total proteome analysis. ORF 04514 is predicted to encode a zinc-containing, NAD^+^-dependent alcohol dehydrogenase (COG1063). A putative gene in direct proximity, ORF 04510, was attributed to prominent spot AS_19, and is annotated to encode a ‘putative redox-active protein’ (PF09719) of unknown function; the identification and induction of this gene during growth with acetone was also confirmed by the total proteome analysis. Gene cluster B contains also two genes for benzoyl-CoA reductase subunits, and genes for enoyl-CoA hydratase, 3-hydroxyacyl-CoA dehydrogenase, and acetyl-CoA acetyltransferase (Figure 3B). The gene for acetyl-CoA acetyltransferase (04509) was co-identified by spot AS_12, however, at much lower score (Table 2). The gene for enoyl-CoA hydratase (04513) was identified with high score for protein spot AS_18 that appeared on both gels, hence, likely represents a constitutively expressed protein during growth with both acetone and butyrate. Notably, all these genes of gene cluster B (Figure 3B) were identified in the total proteome analysis, except for gene 04512, and gene cluster B appears not to be conserved in any other bacterial genome available within the IMG database thus far.

Another prominent, apparently acetone-inducible protein, AS_32 (Figure 1A), corresponded to ORF 01796 which is predicted to encode a desulfoferredoxin (COG2023). This gene is on a contig with predicted genes (01797–01805) that might code for the following proteins: rubrerythrin, hypothetical protein conserved in SRB, cytochrome *bd*-type quinol oxidase subunit 1 and subunit 2, multimeric flavodoxin subunit, rubredoxin, uncharacterized flavoprotein (COG0426), ferredoxin, and DsrE/DsrF-like family protein. However, we could identify only a predicted desulfoferredoxin (01796) as highly expressed protein in our gel-based proteomic approach (Table 2). This complex is similar to the one found in *Desulfovibrio alaskensis* G20 which also contains desulfoferredoxin [23]. The function of the entire complex is not clear, however, it may be involved in the protection of the cells against oxidative stress, e. g., by removing superoxide radicals (see UniProt Q30WF9).

The very prominent, apparently acetone-inducible protein AS_4 (Figure 1A) was encoded by gene 03619 which is predicted to encode the *alpha*-subunit of adenosine phosphosulfate reductase (AprA). However, this enzyme is relevant for both growth conditions since it is involved in the reduction of adenosine phosphosulfate to sulfite and AMP. This gene is located in a cluster with adenosine phosphosulfate reductase *beta*-subunit gene (03620), and also this gene was identified, both in acetone- and butyrate-grown cells, for the very prominent spot AS_23 (and AS_24).

A weak, high-molecular weight band AM_0 appeared in the membrane fraction of acetone-grown cells (Figure 2) and corresponded to gene 04339; it is predicted to encode a CoA-acetylating pyruvate:ferredoxin (flavodoxin) oxidoreductase. Finally, band AM_10 which also appeared to be more prominent in acetone-grown cells than in butyrate-grown cells, corresponded to gene 03042 and is predicted to encode a NADH:ubiquinone oxidoreductase subunit RnfG. This gene is located in a predicted operon with the following genes: NADH: ubiquinone oxidoreductase subunit RnfA (03040); electron transport complex Rnf ABCDGE type, B subunit (03039); electron transport complex Rnf ABCDGE type, C subunit (03044); electron transport complex Rnf ABCDGE type, D subunit (03043); NADH: ubiquinone oxidoreductase RnfE subunit (03041). The total proteome analysis confirmed that genes 04339 and 03042, and, in addition, all other subunit genes of the RnfABCDGE type complex were expressed during growth with acetone and during growth with butyrate (see Additional file 2: Table S1). This Rnf complex might play a role in the supply with low-potential electrons for CO_2_ reduction to provide CO as a co-substrate in the initial carbonylation reaction.

### Proteins identified for growth with both acetone and butyrate

The prominent spot AS_4 (see above; Table 2) corresponded to gene 03619 for the *alpha*-subunit of adenosine phosphosulfate reductase (AprA). This gene is located in a cluster with adenosine phosphosulfate reductase *beta*-subunit gene 03620, and also this gene was identified for the very prominent spots AS_23 and AS_24 (Figure 1AB, Table 3). The gene cluster contains also the gene (03617) for the subunit A of heterodisulfide reductase and related polyferredoxins (HdrA). A similar gene cluster was found in the genomes of *Desulfovibrio vulgaris* DP4, *Desulfovibrio vulgaris* Hildenborough, and *Desulfococcus oleovorans* Hxd3. Spot AS_8 (Figure 1AB) corresponds to gene 04385 which most likely encodes a dissimilatory-type sulfite reductase *alpha*-subunit (Table 3). The gene is co-located in a cluster with the gene for dissimilatory-type sulfite reductase *beta*–subunit (04384), which was also identified by PMF (spot AS_13). In the same cluster, genes are located that code for dissimilatory sulfite reductase D (04383) and for NADH: flavin oxidoreductases of the Old Yellow Enzyme family (04379). This cluster is also found in *Desulfococcus oleovorans* Hxd3, and *Desulfovibrio vulgaris* Hildenborough, and DP4 strains, and in *Desulfovibrio alaskensis* G20.

Also all subunit genes of the ATP synthase complex arranged in a gene cluster in the genome of *D. biacutus* were identified in both acetone- and butyrate-grown cells. These identifications were confirmed by the total proteome analysis.

Band AM_1 (Figure 2) was encoded by ORF 01638 which is annotated as CO dehydrogenase/CO-methylating acetyl-CoA synthase *beta* subunit gene (Table 3). This subunit gene is encoded in a gene cluster together with candidate genes for CO dehydrogenase/acetyl-CoA synthase *delta* subunit (01636), catalytic subunit (01637) and *gamma* subunit (01639), and candidate genes for a pterin binding enzyme (01640), phosphoenolpyruvate synthase/pyruvate phosphate dikinase (01642), and dehydrogenase (01643). The *delta* and *gamma* subunit genes of the CO dehydrogenase complex were identified in the total proteome analysis (see Additional file 1: Table S1). This enzyme complex is used in *D. biacutus* for the oxidation of acetyl residues in the Wood-Ljungdahl pathway.

Band AM_3 (Figure 2) was encoded by ORF 00010 which is annotated as an acetyl-CoA/propionyl-CoA/3-methylcrotonyl-CoA carboxylase/carboxyltransferase *beta* component gene (Table 3); this observation was verified in the total proteome analysis (score for acetone, 1173; score for butyrate, 382). The total proteome analysis identified further genes on the same contig (not shown), for acetyl-CoA acetyltransferase (00008), isopropylmalate/homocitrate/citramalate synthase (00007), acyl-CoA synthetase (AMP-forming)/AMP-acid ligases II (00009), acyl-CoA synthetase (NDP forming) (00014), and methylmalonyl-CoA epimerase (00015), each with a higher score for acetone-grown cells than for butyrate-grown cells (see Additional file 1: Table S1); interestingly, the co-located gene for a sodium-transporting methylmalonyl-CoA decarboxylase (00011) could not be identified. Whether these enzymes are also involved in acetone metabolism or operate solely in amino acid synthesis remains to be elucidated in the future.

## Conclusions

The results of our proteomic analyses clearly document that *D. biacutus* uses a mechanism for acetone activation that is basically different from the carboxylation reactions observed in aerobic or nitrate-reducing bacteria. The genome does not contain genes coding for enzymes that would be comparable to the described acetone carboxylases [1–3]. Rather, our results indicate that acetone is carbonylated in an ATP and TDP-dependent reaction to an aldehyde derivative which is subsequently oxidized to an acetoacetyl-CoA derivative and further to CO_2_. The mechanism of the carbonylation reaction remains unclear at present, including the question whether acetoacetaldehyde is the primary product of this reaction. The acetone-specific synthesis of a cobalamine-containing enzyme indicates that a branched-chain derivative may be formed first which is later isomerized to acetoacetaldehyde. In any case, the novel reaction pathway is energetically more favorable for the energy-deprived metabolism of a sulfate-reducing bacterium: It probably requires one ATP equivalent for acetone activation and a further fraction of an ATP equivalent for CO_2_ reduction to CO employing an Rnf complex to form acetoacetylCoA. The described pathways for acetone degradation by aerobic or nitrate-reducing bacteria require at least 3 ATP equivalents to obtain this intermediate. One may speculate whether the novel concept of substrate carbonylation is applied also in the activation of other comparably stable compounds.

## Methods

### Bacteria and growth conditions

*Desulfococcus biacutus* strain KMRActS (DSM 5651) from our own culture collection was grown in freshwater mineral salts medium in 1 L flasks under an anoxic gas phase N_2_/CO_2_ (80/20) at 30°C in the dark, as described previously [7, 24]. The medium was reduced with sodium sulfide, buffered with bicarbonate and adjusted to pH 7.2, and supplemented with 5 mM acetone or 5 mM butyrate as sole organic carbon source, plus 10 mM sulfate as the electron acceptor.

### DNA extraction, sequencing and annotation

Total DNA was extracted according to the CTAB Protocol for Bacterial DNA extraction of the DoE-JGI (http://my.jgi.doe.gov) including RNAse treatment. Library preparation, sequencing, and assembly were conducted under contract at GATC Biotech AG (Konstanz, Germany). Briefly, two different libraries were prepared, a 3 kb paired-end library and a shotgun library with an insert size of approximately 1800 bp. Both libraries were sequenced using the Roche GS FLX + instrument. The paired-end library was sequenced on 2x 1/16 GS FLX Pico-Titer Plate with GS FLX Titanium XLR70 chemistry, and the shotgun library on 1/2 GS FLX Pico-Titer Plate with GS FLX Titanium XL + chemistry. Analysis of the readout and *de novo* assemblies were done with the GS FLX System Software GS De Novo Assembler (Newbler) version 2.6, using the default parameters and the “read flowgrams” (SFF files) of the shotgun library and the 3 kb paired end library as input. The assembly (153 contigs) was submitted to the IMG annotation pipeline, and the obtained annotation was used to set up a local PMF database at the Proteomics Center of the University of Konstanz.

### Preparation of cell-free extracts

*D. biacutus* cells were collected in the late exponential growth phase (OD_600nm_ ≈ 0.3) by centrifugation (6,000 × *g*, 20 min, 4°C) and washed three times in Tris–HCl buffer (30 mM, pH 7.2). The cell pellet was resuspended in Tris–HCl buffer (30 mM, pH 7.2) supplemented with 0.5 mg DNase mL^−1^ and 1 mg mL^−1^ of Protease Inhibitor Cocktail (Complete Mini tablets, Roche Diagnostics GmbH, Mannheim, Germany), and cells were disrupted by three passages through a chilled French pressure cell (100 MPa; Aminco). Cell debris was removed by centrifugation (27,000 × *g*, 20 min, 4°C) and the membrane fragments collected by ultracentrifugation (50,000 × *g*, 60 min, 4°C); the supernatant was called the soluble protein fraction. Membrane fragments were washed four times with the same buffer and solubilized with a specific phosphate buffer (see below).

### Protein gel electrophoresis

2D-PAGE of soluble proteins was carried out essentially according to our previously published protocol [25] using the BioRad Ready Strip IPG/Protean II system. Briefly, the soluble protein fraction was desalted by gel filtration (Sephadex™ G-25 columns, GE Healthcare) and each sample of 1 mg total protein was precipitated by addition of 5 volumes of ice-cold acetone (overnight, -20°C). The protein precipitate was collected by centrifugation (13,000 × *g*, 10 min, 4°C), the protein pellet air-dried (30 min, RT), the proteins solubilised in rehydration buffer (300 μL) and loaded onto an isoelectric focusing (IEF) strip (BioRad IPG strips, 17 cm, pH 5–8). The isoelectric focusing program involved a voltage ramp (rapid) to a maximal voltage of 10,000 V during 3 h, and a total focusing of 40,000 Volt-hours (Vh). Strips were equilibrated in SDS-equilibration buffers I and II (with DTT and iodoacetamide, respectively) and placed onto an SDS-PAGE gel [26] using an overlay of SDS-gel buffer solidified with agarose (0.5%). The gels (17 × 20 cm; BioRad Protean II XI cell) contained a gradient of 5 - 18% polyacrylamide in the resolving gel and 4% polyacrylamide in the stacking gel. Gels were stained with colloidal Coomassie blue R-250 [27]. Protein spots of interest were excised from the gels and submitted to peptide-fingerprinting-mass spectrometry.

1D-PAGE of membrane proteins involved solubilization of the washed membrane fragments (see above) in 200 mM NaH_2_PO_4_ buffer, pH 6.0, supplemented with 150 mM NaCl and 10% SDS (w/v), incubation of the protein samples with 2 volumes SDS-gel loading buffer with 5% *β*-mercaptoethanol at 100°C for 5 min, prior to loading the samples onto a SDS-PAGE gel; the gels (17 × 20 cm) contained a gradient of 5 - 18% polyacrylamide in the resolving gel and 4% polyacrylamide in the stacking gel, and were stained with colloidal Coomassie blue R-250 (see above). Protein bands of interest were excised from the gels and submitted to peptide-fingerprinting-mass spectrometry.

For a total proteome analysis of crude extracts (soluble and membrane proteins) from acetone and butyrate grown cells, samples with each 30 μg of total protein were mixed with 5% *β*-mercaptoethanol, boiled for 5 min, and loaded onto a small SDS-PAGE gel (8 × 6 cm, BioRad Protean Mini cell), which contained 4% polyacrylamide in the stacking gel and 10% polyacrylamide in the resolving gel. The gel was run until the bands had just entered the resolving gel. The gel was stained with Coomassie blue R-250 (see above), and each band (app. 3 × 3 mm) excised and submitted to high-resolution (Orbitrap) peptide fingerprinting-mass spectrometry.

### Peptide fingerprinting-mass spectrometry and database searching

Protein bands or spots excised from the gels were analyzed at the Proteomics Facility of the University of Konstanz (http://www.proteomics-facility.uni-konstanz.de). All tryptic digests were analyzed by reversed-phase liquid chromatography tandem mass spectrometry (LC-MS/MS) using an Esquire 3000 mass spectrometer (Bruker Daltonics), connected to an Agilent 1100 HPLC. After sample injection, the column was washed for 5 min with 90% mobile phase A (0.1% formic acid) and 10% mobile phase B (0.1% formic acid in acetonitrile), and peptides were eluted using a linear gradient of 10% mobile phase B to 80% mobile phase B in 20 min at 50 μl min^−1^. The Esquire mass spectrometer was operated in a data-dependent mode in which each full MS scan was followed by three MS/MS scans where the three most abundant molecular ions were dynamically selected and fragmented by collision-induced dissociation (CID). Dynamic exclusion was allowed.

The MASCOT engine (Matrix Science, London, UK) was used to match each peptide fingerprint against a local database of all predicted protein sequences of the annotated genome of *Desulfococcus biacutus* KMRAcS (IMG, see above), and against the external EMBL and NCBI databases. The parameters for searching and scoring were as follows. One missed cleavage site allowed. Fixed modifications: carbamidomethyl Cys. Variable modifications: N-term. pyro-Glu, N-term. Gln, Met-oxidation. Peptide charge: 2^+^, 3^+^, 4^+^. Peptide tolerance: 1.0 Da. MS/MS tolerance: 0.8 Da. If not stated otherwise (see Results), a minimal score of 200 and/or minimal sequence coverage of 30% was set as cut-off for low-scoring hits.

### Availability of supporting data

Data supporting the proteomics results are included as Additional file 1: Table S1 and Additional file 2: Table S2) within the article. The draft genome annotation used in this study is available within the IMG platform (https://img.jgi.doe.gov) under IMG Submission ID 7648.

## Electronic supplementary material

Additional file 1: Table S1: Results of the total proteome analysis of crude extract of *D. biacutus* grown with acetone. (PDF 144 KB)

Additional file 2: Table S2: Results of the total proteome analysis of crude extract of *D. biacutus* grown with butyrate. (PDF 161 KB)

## Abbreviations

PMF: Peptide mass fingerprinting
2D-PAGE: Two-dimensional polyacrylamide gel electrophoresis
ORF: Open reading frame.
