# Supplementary material for: Acetone utilization by sulfate-reducing bacteria: draft genome sequence of Desulfococcus biacutus and a proteomic survey of acetone-inducible proteins
Source: BMC Genomics. 2014 Jul 11;15(1):584. doi: 10.1186/1471-2164-15-584 (PMC4103992; doi:10.1186/1471-2164-15-584)
Supplement: Supplementary file 1 — Additional file 1: Table S1: Results of the total proteome analysis of crude extract of D. biacutus grown with acetone. (PDF 144 KB) [file 12864_2014_6264_MOESM1_ESM.pdf]

Additional file for article:

Acetone utilization by sulfate-reducing bacteria: draft genome sequence of *Desulfococcus biacutus* and a proteomic survey of acetone-inducible proteins

Olga B. Gutiérrez Acosta[1,2] David Schleheck[1,2] and Bernhard Schink[1,2]

[1]Department of Biology and [2]Konstanz Research School Chemical Biology,
University of Konstanz, D-78457 Konstanz, Germany

BMC Genomics

Table S1

Results of the total proteome analysis of crude extract of *D. biacutus* grown with acetone.

Table S1. Results of the total proteome analysis of crude extract of *D. biacutus* grown with acetone.

Locus tag	Annotation	Score	Coverage [%]	Peptides	PSMs	AAs	MW [kDa]	calc. pI
DebiaDRAFT_04566	thiamine pyrophosphate-requiring enzymes [acetolactate synthase, pyruvate dehydrogenase (cytochrome), glyoxylate carboligase, phosphonopyruvate decarboxylase]	23485	50	21	1024	685	77,5	6,62
DebiaDRAFT_03619	adenosine phosphosulphate reductase, alpha subunit	18439	57	23	454	657	73,2	7,06
DebiaDRAFT_03586	ATP sulphurylase	13256	49	16	290	423	46,6	6,74
DebiaDRAFT_04339	pyruvate:ferredoxin (flavodoxin) oxidoreductase, homodimeric	7765	47	33	184	1214	131,6	6,20
DebiaDRAFT_02131	chaperonin GroL	7576	58	19	166	548	58,1	5,21
DebiaDRAFT_03345	ATP synthase, F1 beta subunit	6690	68	18	137	471	51,0	5,11
DebiaDRAFT_04514	threonine dehydrogenase and relatedZn-dependent dehydrogenases	6597	54	12	175	355	38,2	6,15
DebiaDRAFT_01637	carbon-monoxide dehydrogenase, catalytic subunit	6194	44	17	155	672	73,3	6,19
DebiaDRAFT_00008	acetyl-CoA acetyltransferases	6190	69	17	186	427	45,7	7,33
DebiaDRAFT_04574	methylmalonyl-CoA mutase, N-terminal domain/subunit	6139	65	17	134	573	64,5	5,20
DebiaDRAFT_01638	CO dehydrogenase/CO-methylating acetyl-CoA synthase complex,	5911	42	20	146	737	80,9	5,73

	beta subunit							
DebiaDRAFT_01639	CO dehydrogenase/acetyl-CoA synthase gamma subunit (corrinoid Fe-S protein)	5428	58	15	137	448	48,1	5,38
DebiaDRAFT_04385	sulfite reductase, dissimilatory-type alpha subunit	5397	56	17	159	437	49,4	5,35
DebiaDRAFT_01636	CO dehydrogenase/acetyl-CoA synthase delta subunit (corrinoid Fe-S protein)	5391	36	11	142	537	56,6	6,18
DebiaDRAFT_03447	NAD(P)H-nitrite reductase	4838	55	18	130	565	60,9	8,12
DebiaDRAFT_00317	acyl-coenzyme A synthetases/AMP-(fatty) acid ligases	4052	57	24	106	588	65,9	6,35
DebiaDRAFT_04384	sulfite reductase, dissimilatory-type beta subunit	3998	28	9	105	382	42,7	7,20
DebiaDRAFT_01815	acyl-coenzyme A synthetases/AMP-(fatty) acid ligases	3959	49	17	88	635	70,1	6,00
DebiaDRAFT_02387	formyltetrahydrofolate synthetase	3738	49	18	100	587	63,6	7,83
DebiaDRAFT_00007	isopropylmalate/homocitrate/citramalate synthases	3562	31	9	87	414	46,6	5,54
DebiaDRAFT_04571	dehydrogenases with different specificities (related to short-chain alcohol dehydrogenases)	3424	61	10	74	264	27,9	6,24
DebiaDRAFT_00009	acyl-CoA synthetases (AMP-forming)/AMP-acid ligases II	3366	41	13	77	523	58,4	5,94
DebiaDRAFT_04163	phospho-2-dehydro-3-deoxyheptonate aldolase	3331	49	9	97	374	40,9	6,27

DebiaDRAFT_02264	aldehyde:ferredoxin oxidoreductase	3030	36	10	71	578	61,0	5,94
DebiaDRAFT_00014	acyl-CoA synthetase (NDP forming)	2833	37	15	65	706	76,6	5,66
DebiaDRAFT_03347	proton translocating ATP synthase, F1 alpha subunit	2737	31	13	66	505	54,7	5,44
DebiaDRAFT_00156	ABC-type branched-chain amino acid transport systems, periplasmic component	2700	36	9	61	388	42,2	5,69
DebiaDRAFT_04513	enoyl-CoA hydratase/carnithine racemase	2644	60	7	58	262	27,6	5,11
DebiaDRAFT_00168	malate dehydrogenase, NAD-dependent	2379	64	11	52	310	32,6	6,57
DebiaDRAFT_02351	5,10-methylene-tetrahydrofolate dehydrogenase/ methenyl tetrahydrofolate cyclohydrolase	2308	41	8	78	303	32,5	8,21
DebiaDRAFT_01781	ABC-type branched-chain amino acid transport systems, periplasmic component	2308	42	9	56	386	40,8	7,52
DebiaDRAFT_02640	catalase/peroxidase HPI	2287	31	14	51	736	81,2	5,38
DebiaDRAFT_04509	acetyl-CoA acetyltransferases	2276	51	9	46	391	41,6	6,09
DebiaDRAFT_03259	acyl-CoA dehydrogenases	2258	28	10	41	604	65,8	5,88
DebiaDRAFT_03620	adenosine phosphosulphate reductase, beta subunit	2205	39	4	42	145	16,2	5,01
DebiaDRAFT_03617	heterodisulfide reductase, subunit A and related polyferredoxins	2195	39	18	61	778	85,1	5,10

DebiaDRAFT_00806	hydroxylamine reductase	2030	42	13	56	542	58,3	6,43
DebiaDRAFT_01454	methylmalonyl-CoA mutase C-terminal domain/ methylmalonyl-CoA mutase N-terminal domain	2005	33	13	51	713	77,9	5,54
DebiaDRAFT_00323	phosphopyruvate hydratase	1942	40	9	36	425	45,1	4,67
DebiaDRAFT_01640	pterin binding enzyme.	1916	40	6	54	308	34,1	4,97
DebiaDRAFT_01450	pyruvate/oxaloacetate carboxyltransferase	1824	32	13	48	679	74,2	6,39
DebiaDRAFT_04510	putative redox-active protein (C_GCAxxG_C_C).	1790	47	6	41	187	20,7	5,41
DebiaDRAFT_02642	peroxiredoxin	1787	51	5	40	132	14,5	5,57
DebiaDRAFT_04573	methylmalonyl-CoA mutase C-terminal domain	1748	57	5	27	136	15,0	5,82
DebiaDRAFT_00182	translation elongation factor EF-G	1717	33	14	46	691	75,5	5,20
DebiaDRAFT_02996	ATPases with chaperone activity, ATP-binding subunit	1636	26	15	42	949	104,5	6,14
DebiaDRAFT_04190	rubrerythrin	1607	43	6	32	173	19,7	6,13
DebiaDRAFT_02452	glutamine synthetase, type I	1601	32	7	38	472	52,5	5,43
DebiaDRAFT_02722	ABC-type amino acid transport/signal transduction systems, periplasmic component/domain	1585	57	10	42	341	36,4	7,08

DebiaDRAFT_00215	hypothetical protein	1549	18	7	37	627	66,4	4,36
DebiaDRAFT_00177	acetate-CoA ligase	1528	23	10	35	675	75,6	6,51
DebiaDRAFT_04718	electron transfer flavoprotein, alpha subunit	1452	24	5	27	319	33,2	5,02
DebiaDRAFT_01822	aspartate/tyrosine/aromatic aminotransferase	1324	30	7	28	442	49,7	7,53
DebiaDRAFT_04168	3-phosphoshikimate 1-carboxyvinyltransferase	1275	38	9	30	419	44,5	6,20
DebiaDRAFT_04164	3-dehydroquinate synthase	1224	29	7	28	342	37,3	6,44
DebiaDRAFT_03346	ATP synthase, F1 gamma subunit	1222	42	8	27	297	32,2	6,15
DebiaDRAFT_00010	acetyl-CoA carboxylase, carboxyltransferase component (subunits alpha and beta)	1174	25	6	27	517	56,6	6,49
DebiaDRAFT_03727	uncharacterized flavoproteins	1144	24	3	29	148	16,2	7,37
DebiaDRAFT_01174	pyruvate, phosphate dikinase	1138	20	13	33	911	100,8	5,30
DebiaDRAFT_04325	NADH:flavin oxidoreductases, Old Yellow Enzyme family	1102	31	10	24	636	68,3	7,27
DebiaDRAFT_01790	acyl-CoA synthetases (AMP-forming)/AMP-acid ligases II	1042	22	8	25	559	61,5	5,92
DebiaDRAFT_01985	TRAP-type C4-dicarboxylate transport system, periplasmic component	1007	32	5	22	343	37,6	7,96

DebiaDRAFT_04515	benzoyl-CoA reductase/2-hydroxyglutaryl-CoA dehydratase subunit, BcrC/BadD/HgdB	949	42	10	37	442	50,8	6,27
DebiaDRAFT_00528	outer membrane receptor proteins, mostly Fe transport	926	17	7	22	737	82,5	4,81
DebiaDRAFT_01727	ketol-acid reductoisomerase	922	33	6	16	350	38,3	5,40
DebiaDRAFT_02701	ABC-type dipeptide transport system, periplasmic component	920	17	6	25	532	59,5	5,85
DebiaDRAFT_03404	methylmalonyl-CoA mutase C-terminal domain	911	16	12	26	1093	121,6	5,53
DebiaDRAFT_03836	alanine dehydrogenase	904	34	8	26	370	39,4	6,86
DebiaDRAFT_03844	DsrE/DsrF-like family.	895	38	2	17	77	8,7	5,83
DebiaDRAFT_01937	uncharacterized Fe-S center protein	888	23	5	18	368	39,5	6,60
DebiaDRAFT_01449	succinyl-CoA synthetase, beta subunit	886	27	8	23	388	41,8	5,96
DebiaDRAFT_01452	acetyl-CoA carboxylase, carboxyltransferase component (subunits alpha and beta)	878	26	9	20	517	55,9	6,19
DebiaDRAFT_04669	thiamine biosynthesis protein ThiC	874	22	5	22	425	46,0	6,48
DebiaDRAFT_00527	putative methyltransferase, YaeB/AF_0241 family	846	38	4	17	181	20,5	7,46
DebiaDRAFT_01784	enoyl-CoA hydratase/carnithine racemase	843	31	4	19	262	27,5	5,20

DebiaDRAFT_03348	ATP synthase, F1 delta subunit	832	46	6	21	183	20,3	6,89
DebiaDRAFT_03049	D-3-phosphoglycerate dehydrogenase	830	29	8	20	530	57,0	5,72
DebiaDRAFT_03845	acyl-CoA synthetases (AMP-forming)/AMP-acid ligases II	808	22	6	15	539	59,7	6,23
DebiaDRAFT_04095	FAD dependent oxidoreductase.	803	25	7	21	446	49,7	5,34
DebiaDRAFT_00350	pyridoxal-phosphate dependent TrpB-like enzyme	800	35	8	21	458	50,3	6,42
DebiaDRAFT_01884	transketolase, bacterial and yeast	796	13	6	18	674	72,4	6,10
DebiaDRAFT_01599	acetyl-CoA acetyltransferases	796	20	4	17	429	45,5	7,02
DebiaDRAFT_00667	hypothetical protein	783	43	3	15	107	11,7	8,63
DebiaDRAFT_01820	acyl-coenzyme A synthetases/AMP-(fatty) acid ligases	773	22	10	26	634	70,1	6,80
DebiaDRAFT_04161	hypothetical protein	769	16	5	16	470	53,2	6,02
DebiaDRAFT_00091	glutamate synthase domain 2	761	28	9	22	550	59,4	7,66
DebiaDRAFT_00096	ATPases involved in chromosome partitioning	759	24	4	17	279	29,9	5,39
DebiaDRAFT_04533	glyceraldehyde-3-phosphate dehydrogenase, type I	742	37	7	28	334	35,5	7,31
DebiaDRAFT_01614	sulfur relay protein, TusE/DsrC/DsvC family	738	26	3	19	105	12,0	5,52

DebiaDRAFT_02444	thiamine biosynthesis protein ThiC	730	13	4	16	439	49,0	6,67
DebiaDRAFT_04113	phosphoenolpyruvate carboxykinase (ATP)	692	18	6	18	547	60,9	6,64
DebiaDRAFT_01169	uncharacterized NAD(FAD)-dependent dehydrogenases	687	26	7	30	566	62,4	6,21
DebiaDRAFT_04377	pyridoxal 5''-phosphate synthase, synthase subunit Pdx1	669	17	3	14	289	31,1	5,95
DebiaDRAFT_03485	anaerobic dehydrogenases, typically selenocysteine-containing	663	16	6	14	753	80,6	6,28
DebiaDRAFT_02783	Peroxiredoxin	663	45	4	13	171	18,5	5,40
DebiaDRAFT_03726	carbon-monoxide dehydrogenase, catalytic subunit	656	15	7	16	658	72,3	6,54
DebiaDRAFT_00534	cobalamin biosynthesis protein CbiK, Co2+ chelatase	651	33	5	15	300	32,5	7,12
DebiaDRAFT_03258	3-hydroxyacyl-CoA dehydrogenase	647	15	2	13	286	30,7	6,25
DebiaDRAFT_03834	FOG: GAF domain	636	16	4	15	349	38,7	6,28
DebiaDRAFT_03057	vacuolar-type H(+)-translocating pyrophosphatase	620	20	6	16	669	68,6	5,76
DebiaDRAFT_00963	succinate-semialdehyde dehydrogenase	613	22	6	16	485	51,2	5,48
DebiaDRAFT_01369	tripartite ATP-independent periplasmic transporter solute receptor, DctP family	606	28	5	17	332	36,7	8,70

DebiaDRAFT_04516	benzoyl-CoA reductase/2-hydroxyglutaryl-CoA dehydratase subunit, BcrC/BadD/HgdB	601	24	5	19	385	43,8	6,37
DebiaDRAFT_04717	electron transfer flavoprotein, beta subunit	600	39	5	19	228	24,1	4,61
DebiaDRAFT_02948	predicted flavin-nucleotide-binding protein structurally related to pyridoxine 5''-phosphate oxidase	581	41	3	14	135	15,0	8,27
DebiaDRAFT_04126	ADP-ribosylglycohydrolase	580	22	5	12	361	38,9	5,66
DebiaDRAFT_02865	aspartate-semialdehyde dehydrogenase, gamma-proteobacterial	571	35	8	15	372	41,2	7,37
DebiaDRAFT_00179	cytidylate kinase	570	18	3	11	210	23,9	8,05
DebiaDRAFT_01913	NADPH-dependent FMN reductase.	564	13	2	12	235	26,0	8,32
DebiaDRAFT_01694	putative Ser protein kinase	559	13	7	16	686	78,1	6,02
DebiaDRAFT_02992	YfdX protein.	558	26	6	15	304	32,2	9,00
DebiaDRAFT_00113	anthranilate phosphoribosyltransferase	557	12	3	11	338	35,7	6,55
DebiaDRAFT_01436	outer membrane protein and related peptidoglycan-associated (lipo)proteins	555	46	6	15	315	34,0	4,86
DebiaDRAFT_04338	hydro-lyases, Fe-S type, tartrate/fumarate subfamily, beta region	548	15	5	11	535	58,7	6,86
DebiaDRAFT_01649	3-methyl-2-oxobutanoate hydroxymethyltransferase	545	24	4	11	280	29,9	6,32

DebiaDRAFT_01605	3-phosphoglycerate kinase	542	19	4	15	399	42,1	6,34
DebiaDRAFT_02746	acyl dehydratase	528	11	1	10	141	15,1	7,43
DebiaDRAFT_00804	NAD(P)H-nitrite reductase	523	40	6	10	220	23,7	8,59
DebiaDRAFT_04015	isocitrate dehydrogenase, NADP-dependent, prokaryotic type	518	13	3	10	407	44,1	5,67
DebiaDRAFT_03712	enoyl-CoA hydratase/carnithine racemase	514	42	6	15	238	27,0	5,82
DebiaDRAFT_03931	NAD-dependent aldehyde dehydrogenases	506	24	6	10	513	54,6	6,62
DebiaDRAFT_04048	universal stress protein UspA and related nucleotide-binding proteins	496	23	2	10	154	17,8	9,33
DebiaDRAFT_03386	fructose/tagatose bisphosphate aldolase	495	15	3	9	424	45,6	6,13
DebiaDRAFT_04392	alcohol dehydrogenase, class IV	489	18	5	14	388	40,9	6,68
DebiaDRAFT_04268	ATP sulphurylase/adenylylsulfate kinase (apsK)	476	13	5	11	571	64,0	7,05
DebiaDRAFT_02147	ribosomal protein S2, bacterial type	474	42	7	16	293	33,2	5,31
DebiaDRAFT_02375	pyruvate:ferredoxin oxidoreductase and related 2-oxoacid:ferredoxin oxidoreductases, beta subunit	442	16	3	8	251	27,6	8,76
DebiaDRAFT_00648	NADPH:quinone reductase and related Zn-dependent oxidoreductases	438	16	2	9	325	34,7	6,86

DebiaDRAFT_03297	threonine dehydrogenase and related Zn-dependent dehydrogenases	437	24	5	11	343	36,6	6,24
DebiaDRAFT_02445	nicotinate-nucleotide dimethylbenzimidazole phosphoribosyltransferase	432	14	3	11	352	36,6	5,54
DebiaDRAFT_00097	predicted DNA-binding proteins	427	35	3	7	137	15,5	8,15
DebiaDRAFT_00359	thioredoxin	415	33	2	7	108	11,7	4,88
DebiaDRAFT_02555	type VI secretion ATPase, ClpV1 family	409	8	4	9	879	96,1	5,49
DebiaDRAFT_01843	5,10-methylenetetrahydrofolate reductase	403	24	5	11	313	34,8	7,87
DebiaDRAFT_03060	NADPH-dependent glutamate synthase beta chain and related oxidoreductases	397	7	4	10	691	74,3	5,85
DebiaDRAFT_00810	response regulator containing CheY-like receiver, AAA-type ATPase, and DNA-binding domains	397	37	3	9	148	16,6	7,34
DebiaDRAFT_02559	type VI secretion system effector, Hcp1 family	397	23	3	13	163	17,4	5,39
DebiaDRAFT_03618	hypothetical protein	395	14	5	10	425	46,4	6,67
DebiaDRAFT_01442	uncharacterized anaerobic dehydrogenase	394	12	3	12	377	41,6	8,15
DebiaDRAFT_03592	citrate synthase I (hexameric type)	393	13	4	9	426	48,5	7,01
DebiaDRAFT_01539	ribosomal protein L14, bacterial/organelle	392	21	2	7	122	13,4	10,20

DebiaDRAFT_00964	anaerobic dehydrogenases, typically selenocysteine-containing	392	7	4	11	691	77,1	7,66
DebiaDRAFT_03195	electron transfer flavoprotein, alpha subunit	385	18	3	7	323	33,3	5,12
DebiaDRAFT_04671	uncharacterized enzyme of thiazole biosynthesis	382	17	3	10	260	27,6	6,38
DebiaDRAFT_03535	molecular chaperone (small heat shock protein)	376	42	4	11	153	17,0	9,69
DebiaDRAFT_00121	leucyl-tRNA synthetase, eubacterial and mitochondrial family	375	7	4	8	863	98,5	6,15
DebiaDRAFT_02794	putative quinone oxidoreductase, YhdH/YhfP family	373	18	4	9	332	35,2	6,40
DebiaDRAFT_03065	predicted NADH:ubiquinone oxidoreductase, subunit RnfC	373	13	4	9	431	46,3	5,22
DebiaDRAFT_00907	acetyl-CoA acetyltransferases	371	26	5	15	395	41,3	7,43
DebiaDRAFT_00370	carbamoyl-phosphate synthase, large subunit	357	7	5	10	1067	116,8	5,54
DebiaDRAFT_01670	hypothetical protein	355	23	2	7	136	15,0	5,29
DebiaDRAFT_04512	3-hydroxyacyl-CoA dehydrogenase	354	15	2	7	283	31,2	7,77
DebiaDRAFT_02462	uroporphyrin-III C-methyltransferase	351	13	3	11	509	54,8	6,74
DebiaDRAFT_03766	ribosomal protein L21	348	47	2	8	116	12,9	9,41
DebiaDRAFT_01635	acetyl-CoA hydrolase	344	5	2	8	627	69,4	5,83

DebiaDRAFT_03292	acyl-CoA dehydrogenases	341	15	3	8	384	42,6	5,76
DebiaDRAFT_03543	predicted Zn-dependent hydrolases of the beta-lactamase fold	339	20	3	9	208	22,6	5,31
DebiaDRAFT_01765	fructose-6-phosphate aldolase, TalC/MipB family	337	18	3	9	214	23,2	5,30
DebiaDRAFT_03805	short-chain dehydrogenases of various substrate specificities	336	18	4	10	266	28,8	5,34
DebiaDRAFT_01783	coenzyme F390 synthetase	325	17	5	9	433	48,3	6,87
DebiaDRAFT_01533	ribosomal protein S5, bacterial/organelle type	324	16	2	8	167	17,6	10,04
DebiaDRAFT_01791	acyl-CoA synthetases (AMP-forming)/AMP-acid ligases II	320	11	3	10	536	59,5	6,83
DebiaDRAFT_01550	ribosomal protein S10, bacterial/organelle	316	17	1	6	103	11,7	9,45
DebiaDRAFT_02374	pyruvate:ferredoxin oxidoreductase and related 2-oxoacid:ferredoxin oxidoreductases, alpha subunit	315	23	3	9	355	38,7	5,27
DebiaDRAFT_00015	methylmalonyl-CoA epimerase	302	28	2	8	143	16,1	5,53
DebiaDRAFT_02364	acyl-CoA dehydrogenases	301	7	2	7	387	42,4	6,80
DebiaDRAFT_02802	aspartate ammonia-lyase	297	12	3	10	470	50,9	5,58
DebiaDRAFT_01814	citrate lyase beta subunit	295	12	3	8	304	32,8	5,82

DebiaDRAFT_02206	NADPH-dependent glutamate synthase beta chain and related oxidoreductases	279	9	3	7	696	74,1	5,12
DebiaDRAFT_04367	coenzyme F420-reducing hydrogenase, alpha subunit	279	6	2	5	449	49,8	6,28
DebiaDRAFT_04165	Prephenate dehydratase	278	9	2	8	374	42,4	7,23
DebiaDRAFT_03042	predicted NADH:ubiquinone oxidoreductase, subunit RnfG	275	9	2	6	227	24,1	8,84
DebiaDRAFT_00094	2-oxoglutarate dehydrogenase, E1 component	258	3	2	6	931	104,9	5,97
DebiaDRAFT_01309	nucleoside-diphosphate-sugar epimerases	254	11	3	6	349	38,7	7,14
DebiaDRAFT_00611	NADPH-dependent glutamate synthase beta chain and related oxidoreductases	248	5	3	5	776	85,0	6,49
DebiaDRAFT_04167	shikimate 5-dehydrogenase	245	12	2	5	294	31,5	5,96
DebiaDRAFT_03762	gamma-glutamyl phosphate reductase	241	9	3	7	418	45,3	6,06
DebiaDRAFT_03403	acetyl-CoA acetyltransferases	239	10	2	6	391	41,2	5,81
DebiaDRAFT_01448	succinyl-CoA synthetase, alpha subunit	232	21	3	8	289	29,8	6,98
DebiaDRAFT_03593	7-cyano-7-deazaguanine reductase	231	19	2	5	131	15,1	6,32
DebiaDRAFT_01051	electron transfer flavoprotein, beta subunit	231	12	2	6	256	27,4	5,58

DebiaDRAFT_00697	enoyl-CoA hydratase/carnithine racemase	228	5	1	5	258	28,1	6,38
DebiaDRAFT_03107	acyl-CoA dehydrogenases	228	12	4	10	555	60,2	5,29
DebiaDRAFT_03260	acetyl-CoA acetyltransferases	226	7	2	6	416	43,7	6,98
DebiaDRAFT_00525	ABC-type Co2+ transport system, periplasmic component	225	12	2	6	268	29,7	5,60
DebiaDRAFT_02337	dehydrogenases with different specificities (related to short-chain alcohol dehydrogenases)	220	9	2	6	255	27,6	6,28
DebiaDRAFT_00264	ATP-binding cassette protein, ChvD family	219	5	2	3	561	63,1	5,54
DebiaDRAFT_00863	predicted lipoprotein involved in nitrous oxide reduction	217	9	1	5	164	18,1	8,41
DebiaDRAFT_03788	alcohol dehydrogenase, class IV	215	10	2	4	355	39,2	5,82
DebiaDRAFT_04170	aspartate/tyrosine/aromatic aminotransferase	207	12	3	6	377	40,9	6,95
DebiaDRAFT_04518	CoA-substrate-specific enzyme activase, putative	206	7	1	3	257	27,3	8,16
DebiaDRAFT_02767	malonyl CoA-acyl carrier protein transacylase	203	18	3	5	311	33,7	6,62
DebiaDRAFT_00671	carbon-monoxide dehydrogenase, catalytic subunit	198	8	2	5	626	66,8	6,54
DebiaDRAFT_01176	alanine dehydrogenase	192	9	2	4	301	33,4	5,17

DebiaDRAFT_03350	F0F1-type ATP synthase, subunit b	182	9	1	7	141	16,1	8,63
DebiaDRAFT_03400	acyl-CoA dehydrogenases	182	16	4	6	385	42,3	7,42
DebiaDRAFT_02725	N-methylhydantoinase A/acetone carboxylase, beta subunit	180	6	2	4	602	65,1	6,30
DebiaDRAFT_03779	predicted pyridoxal phosphate-dependent enzyme apparently involved in regulation of cell wall biogenesis	178	6	2	5	395	43,4	6,54
DebiaDRAFT_02189	acetoacetyl-CoA synthase	159	5	3	5	650	72,4	5,83
DebiaDRAFT_02889	isocitrate lyase	158	10	2	6	445	49,6	6,00
DebiaDRAFT_00368	predicted cobalamin binding protein	155	8	1	3	208	21,5	4,81
DebiaDRAFT_03587	acyl-CoA synthetases (AMP-forming)/AMP-acid ligases II	154	5	2	4	557	61,9	5,74
DebiaDRAFT_04486	Superoxide dismutase	147	6	1	3	229	25,1	6,89
DebiaDRAFT_02002	NAD(P)H:quinone oxidoreductase, type IV	142	9	1	2	248	26,3	6,42
DebiaDRAFT_01042	glycine cleavage system protein P (pyridoxal-binding), C-terminal domain	142	7	1	2	484	52,7	6,44
DebiaDRAFT_02890	malate synthase A	140	5	1	4	533	60,0	6,30
DebiaDRAFT_04324	desulfoferrodoxin ferrous iron-binding domain	139	11	1	4	116	13,2	7,66

DebiaDRAFT_02352	N-acetyl-gamma-glutamyl-phosphate reductase, common form	138	3	1	2	345	37,1	6,61
DebiaDRAFT_04253	peptidoglycan-associated lipoprotein	138	19	1	2	178	19,5	5,26
DebiaDRAFT_04429	tryptophanyl-tRNA synthetase	137	12	2	4	328	37,5	6,62
DebiaDRAFT_00994	uncharacterized enzyme involved in biosynthesis of extracellular polysaccharides	137	40	2	4	104	11,8	8,76
DebiaDRAFT_04491	6-phosphofructokinase	137	6	3	4	697	77,8	5,74
DebiaDRAFT_00699	pantoate--beta-alanine ligase	136	9	2	4	272	30,0	6,09
DebiaDRAFT_02144	ribosome recycling factor	135	12	2	5	185	21,3	8,48
DebiaDRAFT_03110	beta-ketoacyl-acyl-carrier-protein synthase II	130	8	2	4	413	43,5	5,60
DebiaDRAFT_03046	glutaredoxin and related proteins	129	15	1	2	95	10,6	5,48
DebiaDRAFT_02292	ABC-type amino acid transport/signal transduction systems, periplasmic component/domain	129	7	2	5	312	34,7	6,27
DebiaDRAFT_02202	FOG: GAF domain	127	12	2	4	186	21,3	5,43
DebiaDRAFT_01810	class III cytochrome C family.	125	8	1	2	245	26,0	7,11
DebiaDRAFT_00775	amidases related to nicotinamidase	125	8	1	3	186	20,8	6,87

DebiaDRAFT_01554	DNA-directed RNA polymerase, beta'' subunit, predominant form	124	2	2	3	1459	162,0	8,24
DebiaDRAFT_04116	pyruvate:ferredoxin oxidoreductase and related 2-oxoacid:ferredoxin oxidoreductases, gamma subunit	122	13	2	4	195	20,4	9,64
DebiaDRAFT_03291	CO dehydrogenase maturation factor	122	10	2	3	249	26,8	4,82
DebiaDRAFT_02690	anti-anti-sigma factor	122	23	2	2	154	17,2	6,81
DebiaDRAFT_03085	diadenosine tetraphosphate (Ap4A) hydrolase and other HIT family hydrolases	120	9	1	2	139	15,4	7,21
DebiaDRAFT_03760	2,3-bisphosphoglycerate-independent phosphoglycerate mutase	119	3	1	3	523	56,9	5,50
DebiaDRAFT_04653	aspartate racemase	117	14	2	6	234	25,6	5,82
DebiaDRAFT_03811	uncharacterized conserved protein	116	3	1	4	355	38,9	5,49
DebiaDRAFT_02688	PAS domain S-box	116	2	1	2	1266	139,3	5,29
DebiaDRAFT_02207	NADH:ubiquinone oxidoreductase, NADH-binding (51 kD) subunit	115	3	1	2	615	67,2	6,68
DebiaDRAFT_04332	NADH:ubiquinone oxidoreductase, NADH-binding (51 kD) subunit	115	3	1	2	582	62,8	6,19
DebiaDRAFT_00331	uncharacterized conserved protein	115	7	2	3	441	49,1	7,15
DebiaDRAFT_04245	ribosomal protein L13, bacterial type	115	9	1	3	144	16,3	10,07

DebiaDRAFT_02376	2-oxoacid:acceptor oxidoreductase, gamma subunit, pyruvate/2-ketoisovalerate family	114	19	3	5	178	19,2	7,97
DebiaDRAFT_01680	NADPH-dependent glutamate synthase beta chain and related oxidoreductases	113	2	1	2	591	64,3	6,73
DebiaDRAFT_02193	acetyl-CoA carboxylase, carboxyltransferase component (subunits alpha and beta)	113	6	2	4	530	57,9	7,30
DebiaDRAFT_00658	ABC-type nitrate/sulfonate/bicarbonate transport systems, periplasmic components	113	13	2	3	313	34,2	6,81
DebiaDRAFT_03700	nucleoside-diphosphate-sugar epimerases	112	4	1	3	340	38,1	7,59
DebiaDRAFT_01526	ribosomal protein S4, bacterial/organelle type	111	17	2	4	143	16,6	9,70
DebiaDRAFT_03290	deacetylases, including yeast histone deacetylase and acetoin utilization protein	110	10	2	4	316	35,7	5,07
DebiaDRAFT_00612	glutamate synthase domain 2	109	2	1	4	544	59,2	8,46
DebiaDRAFT_00446	D-3-phosphoglycerate dehydrogenase	109	5	2	3	530	57,0	6,49
DebiaDRAFT_04044	PAS domain S-box/diguanylate cyclase (GGDEF) domain	108	11	2	3	452	50,6	5,45
DebiaDRAFT_02171	glycyl-tRNA synthetase, tetrameric type, alpha subunit	108	6	1	2	290	33,5	5,49
DebiaDRAFT_02274	5,10-methylenetetrahydrofolate reductase	108	5	1	2	311	34,5	7,05

DebiaDRAFT_01311	predicted homoserine dehydrogenase	107	6	2	4	434	46,8	7,05
DebiaDRAFT_04569	Zn-dependent hydrolases, including glyoxylases	107	5	1	2	231	25,2	5,74
DebiaDRAFT_00696	lysyl-tRNA synthetase (class II)	107	7	3	5	489	55,6	5,38
DebiaDRAFT_03044	electron transport complex, RnfABCDGE type, C subunit	106	3	1	2	454	47,6	8,66
DebiaDRAFT_00013	acetyl/propionyl-CoA carboxylase, alpha subunit	106	7	1	2	160	16,2	6,06
DebiaDRAFT_02328	Uridylate kinase	106	12	2	3	285	32,0	7,15
DebiaDRAFT_03564	conserved protein/domain typically associated with flavoprotein oxygenases, DIM6/NTAB family	106	6	1	2	190	20,8	7,20
DebiaDRAFT_03827	ABC-type dipeptide transport system, periplasmic component	105	4	2	4	551	62,0	6,90
DebiaDRAFT_00017	predicted thioesterase	105	15	1	6	157	17,1	7,85
DebiaDRAFT_04394	acyl-CoA dehydrogenases	104	2	1	4	594	65,1	6,32
DebiaDRAFT_01555	DNA-directed RNA polymerase, beta subunit	104	2	2	5	1377	154,1	5,95
DebiaDRAFT_00615	citrate synthase	103	3	1	2	394	43,7	6,40
DebiaDRAFT_02757	F0F1-type ATP synthase, subunit b	103	17	2	3	151	17,2	6,05

DebiaDRAFT_01835	ribosomal protein S6	103	21	2	4	143	16,0	4,63
DebiaDRAFT_01887	aconitate hydratase 1	103	6	4	5	916	101,4	5,81
DebiaDRAFT_03064	electron transport complex, RnfABCDGE type, D subunit	101	5	1	2	327	34,9	8,37
DebiaDRAFT_00398	cytochrome c peroxidase	100	5	2	3	422	45,5	4,73
DebiaDRAFT_02209	NADH:ubiquinone oxidoreductase 24 kD subunit	99	11	1	3	164	18,0	5,52
DebiaDRAFT_00327	acyl-CoA synthetases (AMP-forming)/AMP-acid ligases II	97	7	2	4	407	44,0	5,30
DebiaDRAFT_01527	30S ribosomal protein S13	97	10	1	2	127	14,1	10,35
DebiaDRAFT_02071	ribosomal protein L19, bacterial type	97	21	2	3	115	13,4	10,70
DebiaDRAFT_04027	universal stress protein UspA and related nucleotide-binding proteins	96	13	1	3	150	17,1	5,82
DebiaDRAFT_03191	NADPH-dependent FMN reductase.	96	5	1	2	288	31,6	8,27
DebiaDRAFT_04322	Fe-S oxidoreductases	94	15	3	4	317	35,3	7,74
DebiaDRAFT_04517	CoA-substrate-specific enzyme activase, putative	94	9	1	2	278	29,1	7,43
DebiaDRAFT_03767	hypothetical protein	93	7	2	3	324	34,9	5,20

DebiaDRAFT_02567	type VI secretion protein, VC_A0114 family	93	3	1	2	461	52,3	6,01
DebiaDRAFT_02063	transcription elongation factor GreA	93	9	1	4	155	17,0	4,86
DebiaDRAFT_00679	GDP-mannose 4,6-dehydratase	92	6	1	1	361	41,5	6,27
DebiaDRAFT_01542	ribosomal protein L16, bacterial/organelle	91	10	1	4	137	15,4	11,08
DebiaDRAFT_00290	cysteinyl-tRNA synthetase	89	6	2	4	469	53,3	6,20
DebiaDRAFT_00649	acyl-CoA synthetase (NDP forming)	88	4	3	4	902	97,1	6,09
DebiaDRAFT_01559	50S ribosomal protein L11	88	14	1	4	140	15,0	9,57
DebiaDRAFT_01669	hypothetical protein	87	4	1	2	225	24,9	6,67
DebiaDRAFT_01602	glycerol-3-phosphate dehydrogenase	87	16	3	4	343	36,9	8,27
DebiaDRAFT_03680	acyl-CoA synthetase (NDP forming)	87	1	1	2	728	78,2	6,23
DebiaDRAFT_02856	saccharopine dehydrogenase and related proteins	87	4	1	3	398	42,8	5,78
DebiaDRAFT_00407	Fe-S oxidoreductase	87	2	1	2	528	58,7	6,68
DebiaDRAFT_02634	response regulator containing CheY-like receiver, AAA-type ATPase, and DNA-binding domains	87	3	1	3	407	45,5	5,50

DebiaDRAFT_01052	glycine cleavage system T protein (aminomethyltransferase)	85	2	1	2	805	89,9	6,44
DebiaDRAFT_01966	uncharacterized proteins, homologs of lactam utilization protein B	85	5	1	2	250	27,2	6,06
DebiaDRAFT_04336	universal stress protein UspA and related nucleotide-binding proteins	84	7	1	3	141	15,5	6,67
DebiaDRAFT_02076	predicted RNA-binding protein (contains KH domain)	83	38	1	2	76	8,3	9,09
DebiaDRAFT_04130	uncharacterized conserved protein	81	21	1	3	103	11,3	4,63
DebiaDRAFT_01500	UDP-N-acetylglucosamine 2-epimerase	81	3	1	2	375	42,5	6,48
DebiaDRAFT_01856	5,10-methenyltetrahydrofolate synthetase	79	11	2	3	195	22,7	7,44
DebiaDRAFT_03295	predicted archaeal sugar kinases	79	7	2	3	404	44,5	7,05
DebiaDRAFT_04260	succinate dehydrogenase or fumarate reductase, flavoprotein subunit, Bacillus subtilis subgroup	78	2	1	2	637	70,9	7,68
DebiaDRAFT_01957	4-aminobutyrate aminotransferase, prokaryotic type	78	7	2	3	434	46,6	7,55
DebiaDRAFT_03344	ATP synthase, F1 epsilon subunit (delta in mitochondria)	78	11	1	2	137	15,1	9,35
DebiaDRAFT_00233	predicted GTPase	77	6	1	2	201	22,9	9,76
DebiaDRAFT_03765	ribosomal protein L27	77	19	1	2	84	9,1	11,02

DebiaDRAFT_00322	CTP synthase	77	3	2	3	554	61,1	6,01
DebiaDRAFT_00803	NADH:flavin oxidoreductases, Old Yellow Enzyme family	77	8	2	2	644	70,0	7,18
DebiaDRAFT_01813	ABC-type tungstate transport system, permease component	77	14	2	3	301	32,8	8,03
DebiaDRAFT_03163	response regulator containing CheY-like receiver, AAA-type ATPase, and DNA-binding domains	76	10	1	2	117	13,2	5,60
DebiaDRAFT_03556	FOG: GAF domain	76	19	2	3	183	20,1	5,10
DebiaDRAFT_04612	phospho-2-dehydro-3-deoxyheptonate aldolase	75	4	1	2	340	36,8	6,80
DebiaDRAFT_00450	arginyl-tRNA synthetase	75	5	2	2	557	61,7	6,05
DebiaDRAFT_01865	hemolysins and related proteins containing CBS domains	74	14	2	2	185	21,0	7,46
DebiaDRAFT_00293	ABC-type transport system involved in Fe-S cluster assembly, ATPase component	74	12	2	3	254	27,1	5,24
DebiaDRAFT_03734	3-hydroxyacyl-CoA dehydrogenase	73	4	1	2	801	87,4	6,62
DebiaDRAFT_01279	pilus retraction protein PilT	73	3	1	1	384	42,6	7,53
DebiaDRAFT_01907	3-deoxy-8-phosphooctulonate synthase	72	5	1	2	271	29,2	6,42
DebiaDRAFT_03039	electron transport complex, RnfABCDGE type, B subunit	72	5	1	2	303	32,0	5,52

DebiaDRAFT_01760	argininosuccinate lyase	72	4	1	1	469	52,1	6,13
DebiaDRAFT_00858	protein-disulfide isomerase	72	5	1	3	284	31,1	6,44
DebiaDRAFT_01462	3-isopropylmalate dehydratase, small subunit	71	9	1	3	163	18,0	6,19
DebiaDRAFT_03558	DsrE/DsrF-like family protein	71	8	1	1	168	18,6	8,09
DebiaDRAFT_02139	phosphoribosylformylglycinamidine (FGAM) synthase, synthetase domain	71	2	1	2	998	108,6	6,18
DebiaDRAFT_00328	hypothetical protein	71	20	2	4	90	9,9	10,51
DebiaDRAFT_01643	glyceraldehyde-3-phosphate dehydrogenase/erythrose-4-phosphate dehydrogenase	70	8	2	3	415	45,7	6,98
DebiaDRAFT_00088	ABC-type dipeptide transport system, periplasmic component	69	2	1	3	528	58,5	6,81
DebiaDRAFT_01725	2-isopropylmalate synthase, bacterial type	68	3	1	1	513	55,6	6,49
DebiaDRAFT_01723	NADPH:quinone reductase and related Zn-dependent oxidoreductases	68	7	2	2	337	36,5	8,43
DebiaDRAFT_01274	hypothetical protein	68	12	1	3	179	18,6	4,75
DebiaDRAFT_02756	F0F1-type ATP synthase, subunit b	66	7	1	2	198	22,5	8,79
DebiaDRAFT_00550	aminopeptidase N, Escherichia coli type	66	1	1	2	887	100,0	5,57

DebiaDRAFT_04334	cyclic nucleotide-binding domain.	66	7	1	2	155	17,3	5,35
DebiaDRAFT_01634	uncharacterized metal-binding protein	65	2	1	2	650	71,2	5,94
DebiaDRAFT_00342	glycine/serine hydroxymethyltransferase	64	4	1	2	423	45,4	7,08
DebiaDRAFT_03382	glutamate N-acetyltransferase/amino-acid acetyltransferase	63	5	1	2	397	41,6	5,38
DebiaDRAFT_01524	ribosomal protein L17	60	20	1	1	190	20,8	9,52
DebiaDRAFT_04221	ADP-ribosylglycohydrolase	60	5	1	2	303	32,7	5,35
DebiaDRAFT_02735	amidophosphoribosyltransferase	59	4	1	1	468	52,0	7,75
DebiaDRAFT_01154	MinD superfamily P-loop ATPase containing an inserted ferredoxin domain	58	4	1	2	283	30,8	6,76
DebiaDRAFT_00093	2-oxoglutarate dehydrogenase complex dihydrolipoamide succinyltransferase (E2 component)	58	6	2	3	437	47,6	5,44
DebiaDRAFT_04005	UDP-glucose pyrophosphorylase	58	2	1	3	462	51,2	6,40
DebiaDRAFT_02454	pyruvate carboxylase	56	24	1	1	71	7,9	4,41
DebiaDRAFT_04172	prephenate dehydrogenase	56	4	1	2	258	28,7	7,11
DebiaDRAFT_03063	electron transport complex, RnfABCDGE type, G subunit	55	8	1	2	197	20,6	7,18

DebiaDRAFT_01503	predicted nucleoside-diphosphate sugar epimerases	54	5	1	1	340	37,7	6,71
DebiaDRAFT_00547	molybdenum ABC transporter, periplasmic molybdate-binding protein	53	5	1	2	263	27,9	9,39
