# Supplementary material for: Acetone utilization by sulfate-reducing bacteria: draft genome sequence of Desulfococcus biacutus and a proteomic survey of acetone-inducible proteins
Source: BMC Genomics. 2014 Jul 11;15(1):584. doi: 10.1186/1471-2164-15-584 (PMC4103992; doi:10.1186/1471-2164-15-584)
Supplement: Supplementary file 2 — Additional file 2: Table S2: Results of the total proteome analysis of crude extract of D. biacutus grown with butyrate. (PDF 161 KB) [file 12864_2014_6264_MOESM2_ESM.pdf]

Additional file for article:

Acetone utilization by sulfate-reducing bacteria: draft genome sequence of *Desulfococcus biacutus* and a proteomic survey of acetone-inducible proteins

Olga B. Gutiérrez Acosta[1,2] David Schleheck[1,2] and Bernhard Schink[1,2]

[1]Department of Biology and [2]Konstanz Research School Chemical Biology,
University of Konstanz, D-78457 Konstanz, Germany

BMC Genomics

Table S2

Results of the total proteome analysis of crude extract of *D. biacutus* grown with butyrate.

Table S2. Results of the total proteome analysis of crude extract of *D. biacutus* grown with butyrate.

Locus tag	Annotation	Score	Coverage [%]	Peptides	PSMs	AAs	MW [kDa]	calc. pI
DebiaDRAFT_03619	adenosine phosphosulphate reductase, alpha subunit	37657	58	24	944	657	73,2	7,06
DebiaDRAFT_03586	ATP sulphurylase	17257	54	17	402	423	46,6	6,74
DebiaDRAFT_04385	sulfite reductase, dissimilatory-type alpha subunit	8542	65	21	286	437	49,4	5,35
DebiaDRAFT_02131	chaperonin GroL	8023	60	18	187	548	58,1	5,21
DebiaDRAFT_04339	pyruvate:ferredoxin (flavodoxin) oxidoreductase, homodimeric	5379	38	28	125	1214	131,6	6,20
DebiaDRAFT_01639	CO dehydrogenase/acetyl-CoA synthase gamma subunit (corrinoid Fe-S protein)	5319	62	16	126	448	48,1	5,38
DebiaDRAFT_03345	ATP synthase, F1 beta subunit	5204	65	18	107	471	51,0	5,11
DebiaDRAFT_01637	carbon-monoxide dehydrogenase, catalytic subunit	5183	42	18	137	672	73,3	6,19
DebiaDRAFT_04384	sulfite reductase, dissimilatory-type beta subunit	5063	37	12	157	382	42,7	7,20
DebiaDRAFT_01638	CO dehydrogenase/CO-methylating acetyl-CoA synthase complex, beta subunit	4903	37	20	135	737	80,9	5,73

DebiaDRAFT_04566	thiamine pyrophosphate-requiring enzymes [acetolactate synthase, pyruvate dehydrogenase (cytochrome), glyoxylate carboligase, phosphonopyruvate decarboxylase]	4828	39	17	164	685	77,5	6,62
DebiaDRAFT_02387	formyltetrahydrofolate synthetase	4726	47	19	124	587	63,6	7,83
DebiaDRAFT_00091	glutamate synthase domain 2	4705	54	19	129	550	59,4	7,66
DebiaDRAFT_03447	NAD(P)H-nitrite reductase	4365	64	20	111	565	60,9	8,12
DebiaDRAFT_00156	ABC-type branched-chain amino acid transport systems, periplasmic component	3688	45	12	79	388	42,2	5,69
DebiaDRAFT_01636	CO dehydrogenase/acetyl-CoA synthase delta subunit (corrinoid Fe-S protein)	3427	41	12	92	537	56,6	6,18
DebiaDRAFT_03617	heterodisulfide reductase, subunit A and related polyferredoxins	3381	52	22	90	778	85,1	5,10
DebiaDRAFT_03259	acyl-CoA dehydrogenases	3085	38	14	81	604	65,8	5,88
DebiaDRAFT_03347	proton translocating ATP synthase, F1 alpha subunit	2685	38	18	73	505	54,7	5,44
DebiaDRAFT_01781	ABC-type branched-chain amino acid transport systems, periplasmic component	2615	52	13	80	386	40,8	7,52
DebiaDRAFT_03141	chaperonin GroL	2589	51	16	68	542	58,0	5,74
DebiaDRAFT_02996	ATPases with chaperone activity, ATP-binding subunit	2575	26	17	67	949	104,5	6,14

DebiaDRAFT_02798	ABC-type branched-chain amino acid transport systems, periplasmic component	2496	48	9	60	378	40,6	6,79
DebiaDRAFT_04392	alcohol dehydrogenase, class IV	2494	31	8	50	388	40,9	6,68
DebiaDRAFT_01454	methylmalonyl-CoA mutase C-terminal domain/ methylmalonyl-CoA mutase N-terminal domain protein	2493	39	16	59	713	77,9	5,54
DebiaDRAFT_03620	adenosine phosphosulphate reductase, beta subunit	2445	42	5	49	145	16,2	5,01
DebiaDRAFT_04514	threonine dehydrogenase and related Zn-dependent dehydrogenases	2424	54	11	85	355	38,2	6,15
DebiaDRAFT_02153	adenylate kinases	2409	45	8	68	220	24,0	8,76
DebiaDRAFT_00317	acyl-coenzyme A synthetases/AMP-(fatty) acid ligases	2238	47	18	57	588	65,9	6,35
DebiaDRAFT_02264	aldehyde:ferredoxin oxidoreductase	2229	33	13	66	578	61,0	5,94
DebiaDRAFT_00806	hydroxylamine reductase	2140	50	16	61	542	58,3	6,43
DebiaDRAFT_04718	electron transfer flavoprotein, alpha subunit	2040	28	6	50	319	33,2	5,02
DebiaDRAFT_01450	pyruvate/oxaloacetate carboxyltransferase	2025	36	17	59	679	74,2	6,39
DebiaDRAFT_02452	glutamine synthetase, type I	1906	36	9	51	472	52,5	5,43
DebiaDRAFT_04325	NADH:flavin oxidoreductases, Old Yellow Enzyme family	1841	33	11	39	636	68,3	7,27

DebiaDRAFT_04490	ABC-type amino acid transport/signal transduction systems, periplasmic component/domain	1836	45	8	45	275	30,9	6,29
DebiaDRAFT_00168	malate dehydrogenase, NAD-dependent	1830	56	10	49	310	32,6	6,57
DebiaDRAFT_02640	catalase/peroxidase HPI	1817	36	19	50	736	81,2	5,38
DebiaDRAFT_01640	pterin binding enzyme.	1750	40	7	58	308	34,1	4,97
DebiaDRAFT_04574	methylmalonyl-CoA mutase, N-terminal domain/subunit	1736	50	14	52	573	64,5	5,20
DebiaDRAFT_04190	rubrerythrin	1719	37	5	42	173	19,7	6,13
DebiaDRAFT_03404	methylmalonyl-CoA mutase C-terminal domain	1704	19	14	45	1093	121,6	5,53
DebiaDRAFT_04163	phospho-2-dehydro-3-deoxyheptonate aldolase	1643	35	7	54	374	40,9	6,27
DebiaDRAFT_04164	3-dehydroquinate synthase	1620	43	10	37	342	37,3	6,44
DebiaDRAFT_04168	3-phosphoshikimate 1-carboxyvinyltransferase	1617	31	8	49	419	44,5	6,20
DebiaDRAFT_04513	enoyl-CoA hydratase/carnithine racemase	1497	38	5	40	262	27,6	5,11
DebiaDRAFT_01452	acetyl-CoA carboxylase, carboxyltransferase component (subunits alpha and beta)	1497	53	15	40	517	55,9	6,19
DebiaDRAFT_01985	TRAP-type C4-dicarboxylate transport system, periplasmic component	1483	32	6	35	343	37,6	7,96

DebiaDRAFT_04377	pyridoxal 5''-phosphate synthase, synthase subunit Pdx1	1417	40	7	32	289	31,1	5,95
DebiaDRAFT_00009	acyl-CoA synthetases (AMP-forming)/AMP-acid ligases II	1393	29	9	33	523	58,4	5,94
DebiaDRAFT_02351	5,10-methylene-tetrahydrofolate dehydrogenase/Methenyl tetrahydrofolate cyclohydrolase	1392	41	8	42	303	32,5	8,21
DebiaDRAFT_03844	DsrE/DsrF-like family.	1239	38	2	28	77	8,7	5,83
DebiaDRAFT_03057	vacuolar-type H(+)-translocating pyrophosphatase	1221	28	8	30	669	68,6	5,76
DebiaDRAFT_03258	3-hydroxyacyl-CoA dehydrogenase	1208	25	5	29	286	30,7	6,25
DebiaDRAFT_04338	hydro-lyases, Fe-S type, tartrate/fumarate subfamily, beta region	1187	28	11	29	535	58,7	6,86
DebiaDRAFT_01135	response regulator containing CheY-like receiver, AAA-type ATPase, and DNA-binding domains	1158	32	3	22	143	16,1	5,95
DebiaDRAFT_04095	FAD dependent oxidoreductase.	1155	28	8	28	446	49,7	5,34
DebiaDRAFT_03346	ATP synthase, F1 gamma subunit	1138	55	10	28	297	32,2	6,15
DebiaDRAFT_04068	phosphoserine aminotransferase	1118	39	9	25	362	40,0	7,15
DebiaDRAFT_02642	peroxiredoxin	1111	51	5	28	132	14,5	5,57
DebiaDRAFT_02130	co-chaperonin GroES (HSP10)	1110	20	3	30	95	10,5	5,34

DebiaDRAFT_01727	ketol-acid reductoisomerase	1088	31	6	22	350	38,3	5,40
DebiaDRAFT_01822	aspartate/tyrosine/aromatic aminotransferase	1078	30	7	23	442	49,7	7,53
DebiaDRAFT_01551	translation elongation factor TU	1071	30	8	31	397	43,8	5,29
DebiaDRAFT_03836	alanine dehydrogenase	1070	43	11	33	370	39,4	6,86
DebiaDRAFT_01614	sulfur relay protein, TusE/DsrC/DsvC family	1069	36	4	28	105	12,0	5,52
DebiaDRAFT_00595	polyribonucleotide nucleotidyltransferase	1059	23	10	29	697	76,2	5,21
DebiaDRAFT_04573	methylmalonyl-CoA mutase C-terminal domain	1052	64	5	17	136	15,0	5,82
DebiaDRAFT_00323	phosphopyruvate hydratase	1040	34	8	22	425	45,1	4,67
DebiaDRAFT_04509	acetyl-CoA acetyltransferases	1035	27	5	27	391	41,6	6,09
DebiaDRAFT_02783	peroxiredoxin	1035	47	4	14	171	18,5	5,40
DebiaDRAFT_00007	isopropylmalate/homocitrate/citramalate synthases	977	19	7	23	414	46,6	5,54
DebiaDRAFT_02997	molecular chaperone (small heat shock protein)	975	46	7	35	189	21,4	5,73
DebiaDRAFT_04113	phosphoenolpyruvate carboxykinase (ATP)	974	18	7	26	547	60,9	6,64

DebiaDRAFT_02560	type VI secretion protein, EvpB/VC_A0108 family	971	28	8	23	498	56,0	5,34
DebiaDRAFT_02172	glycyl-tRNA synthetase, tetrameric type, beta subunit	962	17	8	27	694	76,0	5,92
DebiaDRAFT_01174	pyruvate, phosphate dikinase	873	11	8	23	911	100,8	5,30
DebiaDRAFT_01436	outer membrane protein and related peptidoglycan-associated (lipo)proteins	866	30	5	21	315	34,0	4,86
DebiaDRAFT_01784	enoyl-CoA hydratase/carnithine racemase	852	31	4	18	262	27,5	5,20
DebiaDRAFT_00458	ABC-type amino acid transport/signal transduction systems, periplasmic component/domain	842	24	4	16	266	29,7	7,40
DebiaDRAFT_01554	DNA-directed RNA polymerase, beta'' subunit, predominant form	840	10	11	30	1459	162,0	8,24
DebiaDRAFT_01449	succinyl-CoA synthetase, beta subunit	837	29	8	21	388	41,8	5,96
DebiaDRAFT_02559	type VI secretion system effector, Hcp1 family	834	28	4	25	163	17,4	5,39
DebiaDRAFT_04717	electron transfer flavoprotein, beta subunit	825	39	6	19	228	24,1	4,61
DebiaDRAFT_01558	ribosomal protein L1, bacterial/chloroplast	823	27	5	23	234	25,3	9,50
DebiaDRAFT_04006	branched-chain amino acid aminotransferase, group II	819	37	8	18	354	39,9	7,15
DebiaDRAFT_03348	ATP synthase, F1 delta subunit	817	51	7	22	183	20,3	6,89

DebiaDRAFT_01958	ABC-type branched-chain amino acid transport systems, periplasmic component	813	24	6	16	417	45,4	8,78
DebiaDRAFT_01550	ribosomal protein S10, bacterial/organelle	800	52	4	17	103	11,7	9,45
DebiaDRAFT_02865	aspartate-semialdehyde dehydrogenase, gamma-proteobacterial	787	36	9	24	372	41,2	7,37
DebiaDRAFT_00667	hypothetical protein	777	52	4	21	107	11,7	8,63
DebiaDRAFT_00008	acetyl-CoA acetyltransferases	770	33	9	21	427	45,7	7,33
DebiaDRAFT_03107	Acyl-CoA dehydrogenases	744	19	7	20	555	60,2	5,29
DebiaDRAFT_00359	thioredoxin	739	56	4	13	108	11,7	4,88
DebiaDRAFT_00256	peptidase T-like protein	723	21	6	15	387	41,5	5,27
DebiaDRAFT_00527	putative methyltransferase, YaeB/AF_0241 family	721	43	5	21	181	20,5	7,46
DebiaDRAFT_00368	predicted cobalamin binding protein	715	51	6	19	208	21,5	4,81
DebiaDRAFT_01599	acetyl-CoA acetyltransferases	712	17	4	17	429	45,5	7,02
DebiaDRAFT_00350	pyridoxal-phosphate dependent TrpB-like enzyme	710	31	7	16	458	50,3	6,42
DebiaDRAFT_00096	ATPases involved in chromosome partitioning	703	35	6	19	279	29,9	5,39

DebiaDRAFT_02445	nicotinate-nucleotide--dimethylbenzimidazole phosphoribosyltransferase	694	13	3	22	352	36,6	5,54
DebiaDRAFT_04533	glyceraldehyde-3-phosphate dehydrogenase, type I	691	40	8	25	334	35,5	7,31
DebiaDRAFT_02892	ABC-type amino acid transport/signal transduction systems, periplasmic component/domain	684	25	3	21	248	26,8	7,23
DebiaDRAFT_00014	acyl-CoA synthetase (NDP forming)	684	9	4	15	706	76,6	5,66
DebiaDRAFT_04417	aspartyl-tRNA synthetase, bacterial type	683	16	7	16	597	67,5	5,83
DebiaDRAFT_02002	NAD(P)H:quinone oxidoreductase, type IV	676	16	2	11	248	26,3	6,42
DebiaDRAFT_00532	ABC-type Fe3+-hydroxamate transport system, periplasmic component	660	20	4	15	363	39,8	8,21
DebiaDRAFT_03134	uncharacterized flavoproteins	660	45	5	13	146	15,5	9,07
DebiaDRAFT_03257	acyl-CoA synthetases (AMP-forming)/AMP-acid ligases II	651	22	7	15	547	61,6	6,40
DebiaDRAFT_00264	ATP-binding cassette protein, ChvD family	643	22	10	21	561	63,1	5,54
DebiaDRAFT_00113	anthranilate phosphoribosyltransferase	631	16	4	15	338	35,7	6,55
DebiaDRAFT_01555	DNA-directed RNA polymerase, beta subunit	622	11	10	16	1377	154,1	5,95
DebiaDRAFT_01057	ABC-type branched-chain amino acid transport systems, periplasmic component	622	18	6	20	419	45,7	6,81

DebiaDRAFT_01649	3-methyl-2-oxobutanoate hydroxymethyltransferase	619	24	4	15	280	29,9	6,32
DebiaDRAFT_00700	S-adenosylmethionine synthetase	614	20	4	14	388	42,4	5,74
DebiaDRAFT_04571	dehydrogenases with different specificities (related to short-chain alcohol dehydrogenases)	611	44	7	18	264	27,9	6,24
DebiaDRAFT_03740	ABC-type dipeptide transport system, periplasmic component	608	14	6	19	548	61,1	7,06
DebiaDRAFT_01544	ribosomal protein L22, bacterial type	605	28	2	10	111	12,1	10,74
DebiaDRAFT_02991	YfdX protein.	603	32	7	20	295	31,7	5,20
DebiaDRAFT_00032	saccharopine dehydrogenase and related proteins	602	24	7	21	440	48,4	6,55
DebiaDRAFT_00697	enoyl-CoA hydratase/carnithine racemase	597	13	3	13	258	28,1	6,38
DebiaDRAFT_01843	5,10-methylenetetrahydrofolate reductase	596	27	6	15	313	34,8	7,87
DebiaDRAFT_01790	acyl-CoA synthetases (AMP-forming)/AMP-acid ligases II	593	18	7	16	559	61,5	5,92
DebiaDRAFT_03805	short-chain dehydrogenases of various substrate specificities	590	33	7	19	266	28,8	5,34
DebiaDRAFT_04167	shikimate 5-dehydrogenase	584	20	3	11	294	31,5	5,96
DebiaDRAFT_01913	NADPH-dependent FMN reductase.	583	8	1	12	235	26,0	8,32

DebiaDRAFT_00863	predicted lipoprotein involved in nitrous oxide reduction	576	15	2	18	164	18,1	8,41
DebiaDRAFT_03712	enoyl-CoA hydratase/carnithine racemase	569	29	4	14	238	27,0	5,82
DebiaDRAFT_01455	methylmalonyl-CoA epimerase	565	47	4	13	134	14,5	5,34
DebiaDRAFT_00907	acetyl-CoA acetyltransferases	561	31	6	15	395	41,3	7,43
DebiaDRAFT_00518	acyl-CoA dehydrogenases	554	5	3	17	604	66,0	6,10
DebiaDRAFT_00348	6,7-dimethyl-8-ribityllumazine synthase	550	38	4	13	157	16,6	5,57
DebiaDRAFT_01937	uncharacterized Fe-S center protein	546	18	4	12	368	39,5	6,60
DebiaDRAFT_02163	molecular chaperone, HSP90 family	543	14	5	15	650	73,2	5,36
DebiaDRAFT_04126	ADP-ribosylglycohydrolase	542	23	5	12	361	38,9	5,66
DebiaDRAFT_03726	carbon-monoxide dehydrogenase, catalytic subunit	539	13	7	15	658	72,3	6,54
DebiaDRAFT_01782	ACT domain-containing protein	539	27	3	10	143	15,9	5,27
DebiaDRAFT_03086	bacterial nucleoid DNA-binding protein	530	28	2	8	90	9,6	10,07
DebiaDRAFT_00649	acyl-CoA synthetase (NDP forming)	526	8	5	14	902	97,1	6,09

DebiaDRAFT_01548	50S ribosomal protein L4, bacterial/organelle	526	19	3	9	207	22,8	10,10
DebiaDRAFT_02206	NADPH-dependent glutamate synthase beta chain and related oxidoreductases	526	12	5	13	696	74,1	5,12
DebiaDRAFT_03060	NADPH-dependent glutamate synthase beta chain and related oxidoreductases	525	10	6	17	691	74,3	5,85
DebiaDRAFT_01803	uncharacterized flavoproteins	515	16	5	14	397	44,9	6,60
DebiaDRAFT_03400	acyl-CoA dehydrogenases	508	16	4	15	385	42,3	7,42
DebiaDRAFT_04229	periplasmic serine protease, Do/DeqQ family	506	15	4	9	480	51,3	5,96
DebiaDRAFT_00524	hypothetical protein	505	27	2	8	110	11,7	7,50
DebiaDRAFT_02378	ribosomal protein S1	494	23	7	17	607	67,9	4,87
DebiaDRAFT_03838	ABC-type branched-chain amino acid transport systems, periplasmic component	489	27	5	8	416	45,2	6,47
DebiaDRAFT_03762	gamma-glutamyl phosphate reductase	481	21	6	14	418	45,3	6,06
DebiaDRAFT_01527	30S ribosomal protein S13	480	33	4	15	127	14,1	10,35
DebiaDRAFT_01088	RNA-binding proteins (RRM domain)	475	33	2	9	90	10,0	9,28
DebiaDRAFT_02077	ribosomal protein S16	473	46	3	8	81	9,3	10,15

DebiaDRAFT_03292	acyl-CoA dehydrogenases	473	19	4	13	384	42,6	5,76
DebiaDRAFT_00398	cytochrome c peroxidase	473	9	3	11	422	45,5	4,73
DebiaDRAFT_01549	50S ribosomal protein L3, bacterial	469	35	4	12	210	22,7	9,99
DebiaDRAFT_00525	ABC-type Co2+ transport system, periplasmic component	466	31	5	13	268	29,7	5,60
DebiaDRAFT_02989	hypothetical protein	462	19	5	16	381	43,4	5,83
DebiaDRAFT_01765	fructose-6-phosphate aldolase, TalC/MipB family	460	24	4	13	214	23,2	5,30
DebiaDRAFT_04244	ribosomal protein S9	459	47	4	16	131	14,7	10,70
DebiaDRAFT_03386	fructose/tagatose bisphosphate aldolase	459	12	4	12	424	45,6	6,13
DebiaDRAFT_00116	methylene-tetrahydrofolate reductase C terminal.	453	38	7	18	235	26,1	7,84
DebiaDRAFT_04510	putative redox-active protein (C_GCAxxG_C_C).	452	19	3	14	187	20,7	5,41
DebiaDRAFT_01810	class III cytochrome C family.	451	8	1	9	245	26,0	7,11
DebiaDRAFT_03054	ribose-phosphate pyrophosphokinase	447	13	3	9	313	34,0	5,78
DebiaDRAFT_02802	aspartate ammonia-lyase	447	12	3	17	470	50,9	5,58

DebiaDRAFT_00804	NAD(P)H-nitrite reductase	436	28	5	12	220	23,7	8,59
DebiaDRAFT_03593	7-cyano-7-deazaguanine reductase	429	27	3	10	131	15,1	6,32
DebiaDRAFT_02794	putative quinone oxidoreductase, YhdH/YhfP family	423	17	4	13	332	35,2	6,40
DebiaDRAFT_03126	ABC-type dipeptide transport system, periplasmic component	414	8	3	9	494	54,2	6,00
DebiaDRAFT_00810	response regulator containing CheY-like receiver, AAA-type ATPase, and DNA-binding domains	411	37	3	9	148	16,6	7,34
DebiaDRAFT_01856	5,10-methenyltetrahydrofolate synthetase	410	21	3	11	195	22,7	7,44
DebiaDRAFT_03845	acyl-CoA synthetases (AMP-forming)/AMP-acid ligases II	402	9	3	12	539	59,7	6,23
DebiaDRAFT_03260	acetyl-CoA acetyltransferases	401	7	2	8	416	43,7	6,98
DebiaDRAFT_02757	F0F1-type ATP synthase, subunit b	400	17	2	10	151	17,2	6,05
DebiaDRAFT_01546	ribosomal protein L2, bacterial/organellar	396	24	4	11	275	30,5	10,70
DebiaDRAFT_02555	type VI secretion ATPase, ClpV1 family	396	5	3	14	879	96,1	5,49
DebiaDRAFT_03680	acyl-CoA synthetase (NDP forming)	391	7	4	10	728	78,2	6,23
DebiaDRAFT_00963	succinate-semialdehyde dehydrogenase	384	16	5	10	485	51,2	5,48

DebiaDRAFT_00010	acetyl-CoA carboxylase, carboxyltransferase component (subunits alpha and beta)	383	15	4	9	517	56,6	6,49
DebiaDRAFT_02202	FOG: GAF domain	382	19	3	12	186	21,3	5,43
DebiaDRAFT_01539	ribosomal protein L14, bacterial/organelle	381	21	2	5	122	13,4	10,20
DebiaDRAFT_01448	succinyl-CoA synthetase, alpha subunit	380	21	3	15	289	29,8	6,98
DebiaDRAFT_00370	carbamoyl-phosphate synthase, large subunit	380	7	6	12	1067	116,8	5,54
DebiaDRAFT_02147	ribosomal protein S2, bacterial type	375	16	4	13	293	33,2	5,31
DebiaDRAFT_03401	cupin domain.	374	23	3	11	179	20,3	5,81
DebiaDRAFT_03485	anaerobic dehydrogenases, typically selenocysteine-containing	373	11	5	8	753	80,6	6,28
DebiaDRAFT_01832	ribosomal protein L9	370	25	3	9	151	16,9	8,87
DebiaDRAFT_03536	prephenate dehydratase	362	24	5	10	354	39,5	5,85
DebiaDRAFT_04515	benzoyl-CoA reductase/2-hydroxyglutaryl-CoA dehydratase subunit, BcrC/BadD/HgdB	361	14	4	13	442	50,8	6,27
DebiaDRAFT_01309	nucleoside-diphosphate-sugar epimerases	355	27	7	14	349	38,7	7,14
DebiaDRAFT_02146	translation elongation factor Ts	354	27	3	8	198	21,7	6,10

DebiaDRAFT_04161	hypothetical protein	352	9	3	7	470	53,2	6,02
DebiaDRAFT_02784	tripartite ATP-independent periplasmic transporter solute receptor, DctP family	352	14	3	8	328	37,0	6,29
DebiaDRAFT_00179	cytidylate kinase	351	18	3	7	210	23,9	8,05
DebiaDRAFT_02207	NADH:ubiquinone oxidoreductase, NADH-binding (51 kD) subunit	350	8	3	9	615	67,2	6,68
DebiaDRAFT_03333	phosphotransacetylase	346	11	4	9	469	50,3	6,58
DebiaDRAFT_01311	predicted homoserine dehydrogenase	341	8	3	13	434	46,8	7,05
DebiaDRAFT_01526	ribosomal protein S4, bacterial/organelle type	339	34	4	12	143	16,6	9,70
DebiaDRAFT_00774	sugar phosphate isomerases/epimerases	337	22	5	9	263	29,2	6,14
DebiaDRAFT_03720	nucleoside diphosphate kinase	336	21	1	9	140	15,3	5,54
DebiaDRAFT_01525	DNA-directed RNA polymerase, alpha subunit, bacterial and chloroplast-type	333	27	5	13	339	38,0	4,82
DebiaDRAFT_01533	ribosomal protein S5, bacterial/organelle type	330	36	4	9	167	17,6	10,04
DebiaDRAFT_03169	Fe-S oxidoreductase	326	14	4	11	445	50,6	7,20
DebiaDRAFT_01912	alanine--tRNA ligase	326	4	3	7	874	96,4	6,16

DebiaDRAFT_02071	ribosomal protein L19, bacterial type	326	30	3	8	115	13,4	10,70
DebiaDRAFT_04335	uncharacterized enzyme involved in biosynthesis of extracellular polysaccharides	325	46	3	9	98	11,3	9,41
DebiaDRAFT_03065	predicted NADH:ubiquinone oxidoreductase, subunit RnfC	322	13	4	8	431	46,3	5,22
DebiaDRAFT_02195	Acyl-CoA dehydrogenases	320	12	3	7	382	42,3	6,58
DebiaDRAFT_01152	uncharacterized conserved protein	319	24	2	8	128	13,4	5,69
DebiaDRAFT_01535	ribosomal protein L6, bacterial type	316	15	2	8	179	19,8	9,88
DebiaDRAFT_03049	D-3-phosphoglycerate dehydrogenase	315	18	5	11	530	57,0	5,72
DebiaDRAFT_02701	ABC-type dipeptide transport system, periplasmic component	315	12	4	10	532	59,5	5,85
DebiaDRAFT_03289	CBS-domain-containing membrane protein	312	10	1	4	225	25,4	8,60
DebiaDRAFT_01901	ribosomal subunit interface protein	312	15	2	7	176	20,1	5,73
DebiaDRAFT_04394	acyl-CoA dehydrogenases	310	4	2	10	594	65,1	6,32
DebiaDRAFT_04719	predicted hydrolases or acyltransferases (alpha/beta hydrolase superfamily)	308	15	4	8	385	43,6	5,66
DebiaDRAFT_00696	Lysyl-tRNA synthetase (class II)	306	9	4	9	489	55,6	5,38

DebiaDRAFT_03616	hypothetical protein	305	5	2	9	382	42,1	8,10
DebiaDRAFT_03558	DsrE/DsrF-like family.	302	15	2	7	168	18,6	8,09
DebiaDRAFT_01176	alanine dehydrogenase	298	9	2	9	301	33,4	5,17
DebiaDRAFT_02993	molecular chaperone (small heat shock protein)	296	22	3	10	152	17,1	5,72
DebiaDRAFT_03766	ribosomal protein L21	295	31	1	5	116	12,9	9,41
DebiaDRAFT_01748	UDP-glucose-4-epimerase	291	5	1	5	334	37,0	6,28
DebiaDRAFT_01346	periplasmic protein involved in polysaccharide export	289	7	1	5	184	20,3	8,82
DebiaDRAFT_01041	glycine cleavage system regulatory protein	286	7	1	6	175	19,7	5,15
DebiaDRAFT_01370	uncharacterized conserved protein	285	20	4	8	238	24,6	9,54
DebiaDRAFT_03297	threonine dehydrogenase and related Zn-dependent dehydrogenases	283	4	1	4	343	36,6	6,24
DebiaDRAFT_02080	uncharacterized NAD(FAD)-dependent dehydrogenases	282	9	2	8	453	47,8	7,27
DebiaDRAFT_01783	coenzyme F390 synthetase	282	14	4	10	433	48,3	6,87
DebiaDRAFT_01663	CRISPR-associated protein Cas7/Csd2, subtype I-C/DVULG	275	23	4	8	340	37,6	6,32

DebiaDRAFT_00745	response regulator containing CheY-like receiver, AAA-type ATPase, and DNA-binding domains	272	8	2	7	304	33,5	5,45
DebiaDRAFT_02375	pyruvate:ferredoxin oxidoreductase and related 2-oxoacid:ferredoxin oxidoreductases, beta subunit	271	9	2	5	251	27,6	8,76
DebiaDRAFT_00331	Uncharacterized conserved protein	271	17	6	10	441	49,1	7,15
DebiaDRAFT_01130	ABC-type branched-chain amino acid transport systems, periplasmic component	266	9	2	5	406	44,9	5,91
DebiaDRAFT_04115	indolepyruvate ferredoxin oxidoreductase, alpha subunit	264	6	3	7	623	67,7	6,44
DebiaDRAFT_04387	dihydrodipicolinate synthase	263	23	5	8	351	37,7	6,05
DebiaDRAFT_01537	ribosomal protein L5	258	12	2	7	179	20,3	9,70
DebiaDRAFT_03984	homoserine dehydrogenase	258	10	3	10	438	46,8	5,35
DebiaDRAFT_02880	uncharacterized conserved protein	258	6	1	5	184	20,4	6,37
DebiaDRAFT_01759	argininosuccinate synthase	256	6	2	7	402	45,2	5,85
DebiaDRAFT_00741	spermidine synthase	256	5	1	8	288	32,7	5,85
DebiaDRAFT_03110	beta-ketoacyl-acyl-carrier-protein synthase II	254	11	3	9	413	43,5	5,60

DebiaDRAFT_01557	ribosomal protein L10	254	12	2	8	173	18,9	9,19
DebiaDRAFT_00341	ribose 5-phosphate isomerase B	250	15	2	7	149	16,3	7,30
DebiaDRAFT_04365	cytidylate kinase	243	12	3	7	279	31,6	7,20
DebiaDRAFT_01820	acyl-coenzyme A synthetases/AMP-(fatty) acid ligases	242	6	4	8	634	70,1	6,80
DebiaDRAFT_03273	threonine aldolase	235	5	1	6	365	38,1	6,80
DebiaDRAFT_04653	aspartate racemase	232	18	3	7	234	25,6	5,82
DebiaDRAFT_04253	peptidoglycan-associated lipoprotein	232	13	2	5	178	19,5	5,26
DebiaDRAFT_03135	bacterial nucleoid DNA-binding protein	232	28	2	6	90	9,7	10,10
DebiaDRAFT_01556	ribosomal protein L7/L12	232	10	1	4	128	13,5	4,59
DebiaDRAFT_00123	ribosomal protein S20	229	22	1	6	88	9,7	12,19
DebiaDRAFT_01635	acetyl-CoA hydrolase	228	5	2	5	627	69,4	5,83
DebiaDRAFT_04130	uncharacterized conserved protein	228	56	3	6	103	11,3	4,63
DebiaDRAFT_01154	MinD superfamily P-loop ATPase containing an inserted ferredoxin domain	225	19	4	10	283	30,8	6,76

DebiaDRAFT_01009	valyl-tRNA synthetase	224	6	3	4	888	100,9	5,91
DebiaDRAFT_02328	uridylate kinase	224	18	3	6	285	32,0	7,15
DebiaDRAFT_03380	nucleotide sugar dehydrogenase	223	7	3	10	436	47,5	5,77
DebiaDRAFT_04048	universal stress protein UspA and related nucleotide-binding proteins	223	14	1	4	154	17,8	9,33
DebiaDRAFT_00347	GTP cyclohydrolase II/3,4-dihydroxy-2-butanone 4-phosphate synthase	222	13	4	8	405	44,8	5,67
DebiaDRAFT_00858	protein-disulfide isomerase	219	18	4	7	284	31,1	6,44
DebiaDRAFT_00577	parvulin-like peptidyl-prolyl isomerase	217	6	2	6	350	38,9	8,82
DebiaDRAFT_04516	benzoyl-CoA reductase/2-hydroxyglutaryl-CoA dehydratase subunit, BcrC/BadD/HgdB	217	10	3	7	385	43,8	6,37
DebiaDRAFT_04549	TRAP-type mannitol/chloroaromatic compound transport system, periplasmic component	216	10	2	9	365	39,6	7,81
DebiaDRAFT_00416	phage tail sheath protein FI	215	5	2	7	509	53,9	5,58
DebiaDRAFT_00327	acyl-CoA synthetases (AMP-forming)/AMP-acid ligases II	213	21	5	9	407	44,0	5,30
DebiaDRAFT_04015	isocitrate dehydrogenase, NADP-dependent, prokaryotic type	212	8	2	3	407	44,1	5,67
DebiaDRAFT_02191	cupin domain./Helix-turn-helix.	211	11	2	7	212	23,7	5,67

DebiaDRAFT_00534	cobalamin biosynthesis protein CbiK, Co2+ chelatase	210	19	3	6	300	32,5	7,12
DebiaDRAFT_04334	cyclic nucleotide-binding domain.	210	15	2	4	155	17,3	5,35
DebiaDRAFT_00013	acetyl/propionyl-CoA carboxylase, alpha subunit	209	7	1	4	160	16,2	6,06
DebiaDRAFT_03403	acetyl-CoA acetyltransferases	209	10	2	8	391	41,2	5,81
DebiaDRAFT_00143	predicted RNA-binding protein containing KH domain, possibly ribosomal protein	209	10	1	7	105	11,9	8,48
DebiaDRAFT_04491	6-phosphofructokinase	209	4	2	6	697	77,8	5,74
DebiaDRAFT_03827	ABC-type dipeptide transport system, periplasmic component	209	8	2	5	551	62,0	6,90
DebiaDRAFT_00093	2-oxoglutarate dehydrogenase complex dihydrolipoamide succinyltransferase (E2 component)	208	2	1	9	437	47,6	5,44
DebiaDRAFT_02050	glutaminyl-tRNA synthetase	208	2	1	3	565	64,7	5,99
DebiaDRAFT_01060	ABC-type branched-chain amino acid transport systems, periplasmic component	207	13	4	8	420	46,6	6,46
DebiaDRAFT_03710	nitroreductase	207	6	1	4	180	19,7	6,77
DebiaDRAFT_00615	Citrate synthase	204	7	2	5	394	43,7	6,40

DebiaDRAFT_02444	thiamine biosynthesis protein ThiC	203	10	3	7	439	49,0	6,67
DebiaDRAFT_02461	porphobilinogen deaminase	202	14	4	8	320	34,4	6,74
DebiaDRAFT_03760	2,3-bisphosphoglycerate-independent phosphoglycerate mutase	201	6	2	6	523	56,9	5,50
DebiaDRAFT_02171	glycyl-tRNA synthetase, tetrameric type, alpha subunit	201	11	2	7	290	33,5	5,49
DebiaDRAFT_01791	acyl-CoA synthetases (AMP-forming)/AMP-acid ligases II	200	8	3	6	536	59,5	6,83
DebiaDRAFT_00293	ABC-type transport system involved in Fe-S cluster assembly, ATPase component	199	11	2	6	254	27,1	5,24
DebiaDRAFT_01957	4-aminobutyrate aminotransferase, prokaryotic type	197	6	2	6	434	46,6	7,55
DebiaDRAFT_02168	parvulin-like peptidyl-prolyl isomerase	196	11	3	7	339	38,7	5,45
DebiaDRAFT_04438	DNA binding domain, excisionase family	196	15	3	8	302	34,0	6,14
DebiaDRAFT_01897	triosephosphate isomerase	196	15	2	5	253	27,6	5,69
DebiaDRAFT_04044	PAS domain S-box/diguanylate cyclase (GGDEF) domain	194	4	1	4	452	50,6	5,45
DebiaDRAFT_03085	diadenosine tetraphosphate (Ap4A) hydrolase and other HIT family hydrolases	191	9	1	4	139	15,4	7,21
DebiaDRAFT_01605	3-phosphoglycerate kinase	191	14	3	7	399	42,1	6,34

DebiaDRAFT_03382	glutamate N-acetyltransferase/amino-acid acetyltransferase	190	11	3	9	397	41,6	5,38
DebiaDRAFT_04170	aspartate/tyrosine/aromatic aminotransferase	190	7	2	5	377	40,9	6,95
DebiaDRAFT_02337	dehydrogenases with different specificities (related to short-chain alcohol dehydrogenases)	188	4	1	6	255	27,6	6,28
DebiaDRAFT_02177	bacterial nucleoid DNA-binding protein	186	28	2	5	90	9,7	10,36
DebiaDRAFT_03344	ATP synthase, F1 epsilon subunit (delta in mitochondria)	186	18	2	6	137	15,1	9,35
DebiaDRAFT_01542	ribosomal protein L16, bacterial/organelle	184	10	1	5	137	15,4	11,08
DebiaDRAFT_02548	formate-dependent nitrite reductase, periplasmic cytochrome c552 subunit	179	8	2	5	488	55,1	7,25
DebiaDRAFT_03456	aspartate/tyrosine/aromatic aminotransferase	178	12	3	5	393	42,6	5,66
DebiaDRAFT_03792	protein of unknown function (DUF2950).	178	5	1	5	333	36,0	5,00
DebiaDRAFT_00964	anaerobic dehydrogenases, typically selenocysteine-containing	174	3	2	6	691	77,1	7,66
DebiaDRAFT_02688	PAS domain S-box	174	2	1	2	1266	139,3	5,29
DebiaDRAFT_01442	uncharacterized anaerobic dehydrogenase	172	12	3	7	377	41,6	8,15
DebiaDRAFT_02772	orotate phosphoribosyltransferase	171	24	2	5	185	20,4	8,76

DebiaDRAFT_02630	Response regulator containing CheY-like receiver, AAA-type ATPase, and DNA-binding domains	170	25	2	5	142	15,6	4,94
DebiaDRAFT_02364	acyl-CoA dehydrogenases	168	9	3	7	387	42,4	6,80
DebiaDRAFT_02374	pyruvate:ferredoxin oxidoreductase and related 2-oxoacid:ferredoxin oxidoreductases, alpha subunit	167	17	2	7	355	38,7	5,27
DebiaDRAFT_00453	aspartyl/glutamyl-tRNA(Asn/Gln) amidotransferase, A subunit	165	2	1	5	490	52,5	6,06
DebiaDRAFT_01968	allophanate hydrolase subunit 2	165	13	3	4	327	34,9	6,44
DebiaDRAFT_01813	ABC-type tungstate transport system, permease component	164	9	2	4	301	32,8	8,03
DebiaDRAFT_01815	acyl-coenzyme A synthetases/AMP-(fatty) acid ligases	162	5	3	6	635	70,1	6,00
DebiaDRAFT_03146	imidazole glycerol phosphate synthase, glutamine amidotransferase subunit	161	3	1	3	524	56,6	6,28
DebiaDRAFT_03195	electron transfer flavoprotein, alpha subunit	161	17	4	8	323	33,3	5,12
DebiaDRAFT_04337	peptidyl-prolyl cis-trans isomerase (rotamase) - cyclophilin family	159	9	1	4	174	19,0	7,46
DebiaDRAFT_02189	acetoacetyl-CoA synthase	157	5	3	6	650	72,4	5,83
DebiaDRAFT_02759	ATP synthase, F0 subunit c	150	3	1	6	323	32,8	5,53
DebiaDRAFT_02868	protein-disulfide isomerase	149	5	1	4	297	33,1	6,34

DebiaDRAFT_02209	NADH:ubiquinone oxidoreductase 24 kD subunit	149	11	1	2	164	18,0	5,52
DebiaDRAFT_03914	enoyl-CoA hydratase/carnithine racemase	147	10	2	4	247	26,7	6,54
DebiaDRAFT_00267	glycogen synthase	147	2	1	6	504	57,3	6,10
DebiaDRAFT_04116	pyruvate:ferredoxin oxidoreductase and related 2-oxoacid:ferredoxin oxidoreductases, gamma subunit	147	23	3	4	195	20,4	9,64
DebiaDRAFT_02890	malate synthase A	146	5	1	4	533	60,0	6,30
DebiaDRAFT_03543	predicted Zn-dependent hydrolases of the beta-lactamase fold	146	22	3	5	208	22,6	5,31
DebiaDRAFT_03777	methionyl-tRNA synthetase/ methionyl-tRNA synthetase C-terminal region/beta chain	145	4	2	4	649	73,2	6,81
DebiaDRAFT_00097	predicted DNA-binding proteins	145	24	2	3	137	15,5	8,15
DebiaDRAFT_03734	3-hydroxyacyl-CoA dehydrogenase	144	6	3	6	801	87,4	6,62
DebiaDRAFT_00671	carbon-monoxide dehydrogenase, catalytic subunit	139	8	2	4	626	66,8	6,54
DebiaDRAFT_01643	glyceraldehyde-3-phosphate dehydrogenase /erythrose-4-phosphate dehydrogenase	139	9	2	5	415	45,7	6,98
DebiaDRAFT_04165	prephenate dehydratase	138	4	1	5	374	42,4	7,23

DebiaDRAFT_00775	amidases related to nicotinamidase	137	8	1	3	186	20,8	6,87
DebiaDRAFT_02184	tryptophan synthase, alpha subunit	137	11	2	6	253	27,8	6,10
DebiaDRAFT_03700	nucleoside-diphosphate-sugar epimerases	136	4	1	6	340	38,1	7,59
DebiaDRAFT_00528	outer membrane receptor proteins, mostly Fe transport	135	4	2	5	737	82,5	4,81
DebiaDRAFT_03484	class III cytochrome C family.	134	5	1	4	215	24,1	6,15
DebiaDRAFT_01772	ABC-type uncharacterized transport system, periplasmic component	133	9	2	6	328	35,1	6,35
DebiaDRAFT_02889	isocitrate lyase	133	10	2	5	445	49,6	6,00
DebiaDRAFT_03788	alcohol dehydrogenase, class IV	131	4	1	4	355	39,2	5,82
DebiaDRAFT_03816	cold shock proteins	131	15	1	4	66	7,1	6,52
DebiaDRAFT_03039	electron transport complex, RnfABCDGE type, B subunit	131	5	1	4	303	32,0	5,52
DebiaDRAFT_00354	prolyl-tRNA synthetase, family I	128	5	2	4	506	57,7	6,60
DebiaDRAFT_04677	ABC-type transport system involved in Fe-S cluster assembly, permease component	128	3	1	4	393	43,2	5,57
DebiaDRAFT_04245	ribosomal protein L13, bacterial type	127	9	1	4	144	16,3	10,07

DebiaDRAFT_03042	predicted NADH:ubiquinone oxidoreductase, subunit RnfG	126	9	2	4	227	24,1	8,84
DebiaDRAFT_04452	thioredoxin reductase	125	4	1	4	300	31,8	6,09
DebiaDRAFT_03350	F0F1-type ATP synthase, subunit b	125	9	1	4	141	16,1	8,63
DebiaDRAFT_02724	deacetylases, including yeast histone deacetylase and acetoin utilization protein	124	3	1	4	438	49,8	5,69
DebiaDRAFT_02567	type VI secretion protein, VC_A0114 family	124	3	1	2	461	52,3	6,01
DebiaDRAFT_01052	glycine cleavage system T protein (aminomethyltransferase)	123	5	3	5	805	89,9	6,44
DebiaDRAFT_02144	ribosome recycling factor	123	12	2	4	185	21,3	8,48
DebiaDRAFT_04324	desulfoferrodoxin ferrous iron-binding domain	123	11	1	4	116	13,2	7,66
DebiaDRAFT_00658	ABC-type nitrate/sulfonate/bicarbonate transport systems, periplasmic components	121	20	4	6	313	34,2	6,81
DebiaDRAFT_02139	phosphoribosylformylglycinamidine (FGAM) synthase, synthetase domain	121	2	1	4	998	108,6	6,18
DebiaDRAFT_01602	glycerol-3-phosphate dehydrogenase	119	11	2	5	343	36,9	8,27
DebiaDRAFT_04027	universal stress protein UspA and related nucleotide-binding proteins	114	13	1	4	150	17,1	5,82
DebiaDRAFT_00352	Zn-dependent hydrolases, including glyoxylases	114	8	1	2	206	22,0	6,43

DebiaDRAFT_01669	hypothetical protein	113	4	1	2	225	24,9	6,67
DebiaDRAFT_00561	Fe-S-cluster-containing hydrogenase components 1	112	6	1	2	265	29,6	7,40
DebiaDRAFT_00092	dihydrolipoamide dehydrogenase	111	3	1	2	470	49,7	6,30
DebiaDRAFT_02454	pyruvate carboxylase	111	24	1	2	71	7,9	4,41
DebiaDRAFT_03538	predicted RNA-binding protein (contains KH domain)	110	22	1	2	76	8,3	8,46
DebiaDRAFT_00450	arginyl-tRNA synthetase	110	3	1	2	557	61,7	6,05
DebiaDRAFT_02192	isopropylmalate/homocitrate/citramalate synthases	109	10	1	2	390	44,0	6,60
DebiaDRAFT_01025	phenylalanyl-tRNA synthetase, beta subunit, non-spirochete bacterial	108	5	3	4	807	88,0	5,19
DebiaDRAFT_01559	50S ribosomal protein L11	103	14	1	4	140	15,0	9,57
DebiaDRAFT_00746	response regulator containing CheY-like receiver,<br>AAA-type ATPase, and DNA-binding domains	102	14	2	5	148	16,5	5,87
DebiaDRAFT_03044	electron transport complex, RnfABCDGE type, C subunit	101	3	1	2	454	47,6	8,66
DebiaDRAFT_02767	malonyl CoA-acyl carrier protein transacylase	101	3	1	2	311	33,7	6,62
DebiaDRAFT_01814	citrate lyase beta subunit	100	9	2	4	304	32,8	5,82

DebiaDRAFT_00611	NADPH-dependent glutamate synthase beta chain and related oxidoreductases	100	3	2	3	776	85,0	6,49
DebiaDRAFT_00044	GMP synthase (glutamine-hydrolyzing) domain protein	99	7	3	3	510	56,3	5,82
DebiaDRAFT_00377	benzoyl-CoA reductase/2-hydroxyglutaryl-CoA dehydratase subunit, BcrC/BadD/HgdB	99	6	2	4	425	47,0	5,39
DebiaDRAFT_00701	adenosylhomocysteinase	98	6	2	4	484	53,3	5,76
DebiaDRAFT_04221	ADP-ribosylglycohydrolase	97	19	3	4	303	32,7	5,35
DebiaDRAFT_01835	ribosomal protein S6	95	21	2	4	143	16,0	4,63
DebiaDRAFT_00547	molybdenum ABC transporter, periplasmic molybdate-binding protein	94	9	2	4	263	27,9	9,39
DebiaDRAFT_00813	predicted permeases	94	3	1	2	428	45,1	9,50
DebiaDRAFT_01543	ribosomal protein S3, bacterial type	93	7	1	2	212	24,1	9,96
DebiaDRAFT_03564	conserved protein/domain typically associated with flavoprotein oxygenases, DIM6/NTAB family	92	6	1	2	190	20,8	7,20
DebiaDRAFT_04669	thiamine biosynthesis protein ThiC	92	7	2	4	425	46,0	6,48
DebiaDRAFT_01443	threonyl-tRNA synthetase	92	7	3	4	638	73,2	6,51
DebiaDRAFT_00369	pterin binding enzyme.	91	13	2	2	265	28,7	5,03

DebiaDRAFT_00031	aspartate/tyrosine/aromatic aminotransferase	91	5	2	4	405	45,1	7,28
DebiaDRAFT_03050	domain of unknown function (DUF1844).	90	17	2	3	118	13,3	5,33
DebiaDRAFT_01310	dTDP-4-dehydrorhamnose 3,5-epimerase	90	5	1	4	176	20,7	6,39
DebiaDRAFT_03156	Fe-S oxidoreductase	88	5	2	2	463	52,7	5,80
DebiaDRAFT_03985	phosphoserine phosphatase/homoserine phosphotransferase bifunctional protein	88	10	2	4	203	22,9	5,90
DebiaDRAFT_03349	F0F1-type ATP synthase, subunit b	87	5	1	4	233	25,4	5,92
DebiaDRAFT_01621	predicted metal-dependent hydrolase of the TIM-barrel fold	86	7	2	3	279	31,3	6,35
DebiaDRAFT_04332	NADH:ubiquinone oxidoreductase, NADH-binding (51 kD) subunit	86	3	1	1	582	62,8	6,19
DebiaDRAFT_02355	nucleoside-diphosphate-sugar epimerases	86	4	1	2	326	36,2	6,19
DebiaDRAFT_03063	electron transport complex, RnfABCDGE type, G subunit	84	5	1	2	197	20,6	7,18
DebiaDRAFT_03402	3-hydroxyacyl-CoA dehydrogenase	84	5	1	2	283	31,0	7,02
DebiaDRAFT_02561	type VI secretion protein, VC_A0107 family	82	13	1	2	174	19,7	5,27
DebiaDRAFT_04261	succinate dehydrogenase/fumarate reductase, Fe-S protein subunit	82	4	1	3	255	27,5	6,29

DebiaDRAFT_03787	glucosamine 6-phosphate synthetase, contains amidotransferase and phosphosugar isomerase domains	82	2	2	3	1193	132,1	6,74
DebiaDRAFT_02492	uncharacterized protein conserved in bacteria (DUF2272)./Cell Wall Hydrolase.	81	6	2	2	505	55,0	6,42
DebiaDRAFT_04322	Fe-S oxidoreductases	81	5	1	2	317	35,3	7,74
DebiaDRAFT_04447	ferritin-like protein	80	10	1	3	182	20,8	4,86
DebiaDRAFT_03142	co-chaperonin GroES (HSP10)	80	11	1	2	95	10,5	5,11
DebiaDRAFT_03064	electron transport complex, RnfABCDGE type, D subunit	79	5	1	2	327	34,9	8,37
DebiaDRAFT_04018	uncharacterized protein conserved in bacteria	79	15	1	2	88	9,8	9,69
DebiaDRAFT_01274	hypothetical protein	79	12	1	3	179	18,6	4,75
DebiaDRAFT_04644	transposase	79	13	3	3	322	37,1	9,13
DebiaDRAFT_01933	quinolinate synthetase complex, A subunit	78	9	2	3	338	37,2	5,99
DebiaDRAFT_01461	3-isopropylmalate dehydratase, large subunit	78	3	1	2	418	44,4	5,24
DebiaDRAFT_04361	7-keto-8-aminopelargonate synthetase and related enzymes	77	8	1	2	408	44,1	7,64
DebiaDRAFT_03341	tyrosyl-tRNA synthetase	76	8	3	3	428	47,6	6,27

DebiaDRAFT_03715	aspartate kinase, monofunctional class	76	6	2	3	406	43,5	5,43
DebiaDRAFT_03587	acyl-CoA synthetases (AMP-forming)/AMP-acid ligases II	76	2	1	2	557	61,9	5,74
DebiaDRAFT_03691	predicted hydrolases or acyltransferases (alpha/beta hydrolase superfamily)	75	6	1	2	297	32,1	6,16
DebiaDRAFT_03191	NADPH-dependent FMN reductase.	74	5	1	3	288	31,6	8,27
DebiaDRAFT_01884	transketolase, bacterial and yeast	74	3	2	3	674	72,4	6,10
DebiaDRAFT_03603	NAD-dependent protein deacetylases, SIR2 family	73	4	1	2	274	30,4	7,50
DebiaDRAFT_02194	enoyl-CoA hydratase/carnithine racemase	73	3	1	2	260	28,2	6,76
DebiaDRAFT_02431	ATP phosphoribosyltransferase	72	3	1	2	291	32,5	6,58
DebiaDRAFT_02181	uncharacterized protein, possibly involved in aromatic compounds catabolism	71	21	2	3	127	13,8	6,18
DebiaDRAFT_01168	cyclopropane fatty acid synthase and related methyltransferases	71	6	1	3	289	30,9	5,71
DebiaDRAFT_04034	trimethylamine:corrinoid methyltransferase	71	2	1	3	484	52,1	6,11
DebiaDRAFT_02562	type VI secretion-associated protein, ImpA family	71	9	3	3	440	48,4	4,70
DebiaDRAFT_04477	GYD domain protein	69	10	1	2	124	13,6	5,24

DebiaDRAFT_04336	universal stress protein UspA and related nucleotide-binding proteins	69	7	1	2	141	15,5	6,67
DebiaDRAFT_04172	prephenate dehydrogenase	69	4	1	3	258	28,7	7,11
DebiaDRAFT_04418	histidyl-tRNA synthetase	69	6	2	3	418	46,7	7,09
DebiaDRAFT_01106	hypothetical protein	69	6	1	1	345	38,7	5,67
DebiaDRAFT_03436	aspartate carbamoyltransferase	69	8	2	3	313	35,6	6,67
DebiaDRAFT_01153	MinD superfamily P-loop ATPase containing an inserted ferredoxin domain	68	5	1	2	295	31,5	5,08
DebiaDRAFT_03437	ornithine carbamoyltransferase	67	8	1	2	333	36,7	6,05
DebiaDRAFT_00401	DNA polymerase III, beta subunit	65	4	1	2	371	41,3	4,98
DebiaDRAFT_03109	acyl carrier protein	65	13	1	2	80	9,0	4,15
DebiaDRAFT_01027	ribosomal protein L20	65	20	1	2	116	13,2	11,55
DebiaDRAFT_03290	deacetylases, including yeast histone deacetylase and acetoin utilization protein	64	6	1	2	316	35,7	5,07
DebiaDRAFT_00803	NADH:flavin oxidoreductases, Old Yellow Enzyme family	63	6	2	2	644	70,0	7,18
DebiaDRAFT_00516	predicted RNA-binding protein	60	15	1	2	62	7,1	4,49

DebiaDRAFT_04166	S23 ribosomal protein.	58	18	2	2	119	13,4	8,94
DebiaDRAFT_02376	2-oxoacid:acceptor oxidoreductase, gamma subunit, pyruvate/2-ketoisovalerate family	57	23	3	3	178	19,2	7,97
DebiaDRAFT_00117	class III cytochrome C family.	52	14	1	1	138	15,2	6,70
DebiaDRAFT_02864	3-oxoacyl-[acyl-carrier-protein] synthase III	47	8	1	1	353	39,0	6,29
DebiaDRAFT_03672	molybdopterin converting factor, large subunit	45	12	1	2	121	13,3	8,16
